# Distinct roles of androgen receptor, estrogen receptor alpha, and BCL6 in the establishment of sex-biased DNA methylation in mouse liver

**DOI:** 10.1038/s41598-021-93216-6

**Published:** 2021-07-02

**Authors:** Najla AlOgayil, Klara Bauermeister, Jose Hector Galvez, Varun S. Venkatesh, Qinwei Kim-wee Zhuang, Matthew L. Chang, Rachel A. Davey, Jeffrey D. Zajac, Kinuyo Ida, Akihide Kamiya, Teruko Taketo, Guillaume Bourque, Anna K. Naumova

**Affiliations:** 1 Department of Human Genetics, McGill University, Montréal, QC Canada; 2 Canadian Centre for Computational Genomics, Montréal, QC Canada; 3 Department of Medicine, Austin Health, The University of Melbourne, Heidelberg, VIC 3084 Australia; 4 Department of Biochemistry, McGill University, Montréal, QC Canada; 5 Department of Molecular Life Sciences, Tokai University School of Medicine, 143 Shimokasuya, Isehara, Kanagawa 259-1193 Japan; 6 The Research Institute of the McGill University Health Centre, Montreal, QC H4A 1J3 Canada; 7 Department of Surgery, McGill University, Montreal, QC Canada; 8 Department of Obstetrics and Gynecology, McGill University, Montreal, QC Canada

**Keywords:** Genetics, Molecular biology

## Abstract

Sexual dimorphism in gene regulation, including DNA methylation, is the main driver of sexual dimorphism in phenotypes. However, the questions of how and when sex shapes DNA methylation remain unresolved. Recently, using mice with different combinations of genetic and phenotypic sex, we identified sex-associated differentially methylated regions (sDMRs) that depended on the sex phenotype. Focusing on a panel of validated sex-phenotype dependent male- and female-biased sDMRs, we tested the developmental dynamics of sex bias in liver methylation and the impacts of mutations in the androgen receptor, estrogen receptor alpha, or the transcriptional repressor *Bcl6* gene. True hermaphrodites that carry both unilateral ovaries and contralateral testes were also tested. Our data show that sex bias in methylation either coincides with or follows sex bias in the expression of sDMR-proximal genes, suggesting that sex bias in gene expression may be required for demethylation at certain sDMRs. Global ablation of AR, ESR1, or a liver-specific loss of BCL6, all alter sDMR methylation, whereas presence of both an ovary and a testis delays the establishment of male-type methylation levels in hermaphrodites. Moreover, the *Bcl6*-LKO shows dissociation between expression and methylation, suggesting a distinct role of BCL6 in demethylation of intragenic sDMRs.

## Introduction

DNA methylation plays major roles in gene regulation, genome organization and stability, and silencing of endogenous retroviruses and repeats. Multiple lines of evidence demonstrate that DNA methylation may have yet another function associated with the establishment or maintenance of sexual dimorphism in the epigenome. Sex-biased DNA methylation has been documented in mammals, birds, reptiles, fish, and insects^1–4^. However, its functions and the mechanisms required for its establishment in non-gonadal tissues are still poorly understood.

Sex bias in DNA methylation is cell-type specific and most extensively studied in the mouse liver^5–7^. One of the main findings is the association between sex-biased DNA methylation and sex-biased expression, which is supported by the following observations: (1) sex bias in methylation mirrors sex bias in gene expression^6^; (2) certain mutations that abolish sex bias in gene expression also affect sex-biased methylation; and, finally, (3) the topological proximity between sex-associated differentially methylated regions (sDMRs) and genes with sex-biased expression (referred to from this point on as sex-associated differentially expressed genes, or sDEGs)^6^. Therefore, it is logical to assume that certain cell-type specific regulatory factors are responsible for both phenomena, sex-biased methylation and sex-biased expression.

Our recent whole-genome bisulfite sequencing analysis (WGBS) of the adult mouse liver DNA identified more than 5000 sDMRs across all chromosomes that had 20% or larger difference in methylation levels between males and females^6^. The vast majority of autosomal sDMRs were detected when comparing phenotypic females and males, independent of their sex-chromosome complement, i.e. XX females versus XY males and XY females versus XY males, and therefore were associated with phenotypic sex. Here, we refer to such sDMRs as sex-phenotype dependent. About 90% of autosomal sDMRs have lower methylation in males (referred to as male-biased) and the majority reside in intergenic and intronic regions. sDMR annotation shows enrichment of enhancers, nevertheless more than 50% of sDMRs are associated with quiescent chromatin^6^.

Developmental timing of sex bias in methylation and its relationship with expression offer some clues to the mechanisms involved in the establishment of sex-biased methylation. The vast majority of male-biased sDMRs in the mouse liver appear postnatally around puberty, apparently due to demethylation of certain DNA regions^8^. Castration of prepubescent mice prevents demethylation, whereas supplementation with testosterone at an early age supports the formation of sDMRs^8^. The dynamics of sex-biased methylation is consistent with the dynamics of sex-biased gene expression, where the overwhelming majority of sex-biased genes are detected in adult 8-week old mice, and only about 1% and 14% are detected at 3 and 4 weeks of age, respectively^9^. In addition to testosterone, sex differential secretion patterns of the growth hormone (GH) by the mouse pituitary have been implicated as major drivers of sexual dimorphism in liver biology^10,11^. Several GH-regulated transcription factors (TF) have been proposed as mediators of the sex-specific impact of GH signaling on gene regulation in the mouse liver^12^. For example, mice with liver-specific deletion of signal transducer and activator of transcription 5 (*Stat5a* and *b*) lose sex bias in the expression of nearly 60% of the sDEGs^12,13^. This loss of sex bias in expression is accompanied by hypermethylation of sDMRs that co-localize with STAT5-associated enhancer regions^13^. Another potential downstream effector of GH-signaling, B cell leukemia/lymphoma 6 (BCL6) has been also implicated in sex-biased expression, and *Bcl6* liver-specific knockouts show loss of sex-biased expression in male livers^14^. Liver-specific transcriptional repressor CUX2 with a strong female bias in expression has been also implicated in sex-biased transcriptional regulation^15^. Thus, evidence accumulated to date supports the notion that sDMRs are functionally associated with sex-biased gene expression and hormone signaling pathways. Furthermore, several liver-specific TFs contribute to sex bias in gene regulation.

About 10% of sDMRs have lower methylation in female livers (referred to as female-biased sDMRs), whereas 50% of sex-phenotype dependent DEGs identified in our comparison between sex-reversed XY females and males, have higher expression in females (referred to as female-biased sDEGs)^6^. These numbers are consistent with other groups reporting that the majority of sex-biased sDMRs have lower methylation in males, whereas about half of the sex-biased genes in adult livers have higher expression in females^5,8,9^. However, since to date the main research focus has been on male-biased sDMRs, little is known about the developmental dynamics of female-biased sDMRs, their relationship with female-biased sDEGs, and the role of estradiol signaling in the establishment or maintenance of sex-biased methylation in the mouse liver.

To better understand the relationship between sex-biased methylation and sex-biased expression and the roles of receptors of sex steroid hormones, here, we focused on sDMRs that depended on the sex phenotype rather than sex-chromosome complement. We tested the age dynamics of both female- and male-biased sex-phenotype dependent sDMRs in the livers of male, female, and XY hermaphrodite mice. We also tested the impacts of mutations in genes involved in testosterone, estradiol, or GH-signaling, i.e. androgen receptor (*Ar*), estrogen receptor alpha (*Esr1*), and *Bcl6*, on DNA methylation. We find that all three mutations modify DNA methylation at sDMRs. We also find that at certain sDMRs, sex-biased expression precedes sex-biased methylation.

## Results

### Dynamics of sex-biased methylation and sex-biased expression in mouse liver

One of the challenges in studying the relationship between sDMRs and sDEGs is the difficulty of identifying sDMRs that are functionally associated with specific sDEGs. The possibility of long-range interactions between distant promoters and enhancers located hundreds of kilobases apart is well documented, hence, in principle, a regulatory sDMR may be quite far from its *cis*-regulated gene. Moreover, genes located within the same topologically associated domain may be regulated by shared regulatory elements.

**Figure 1:**
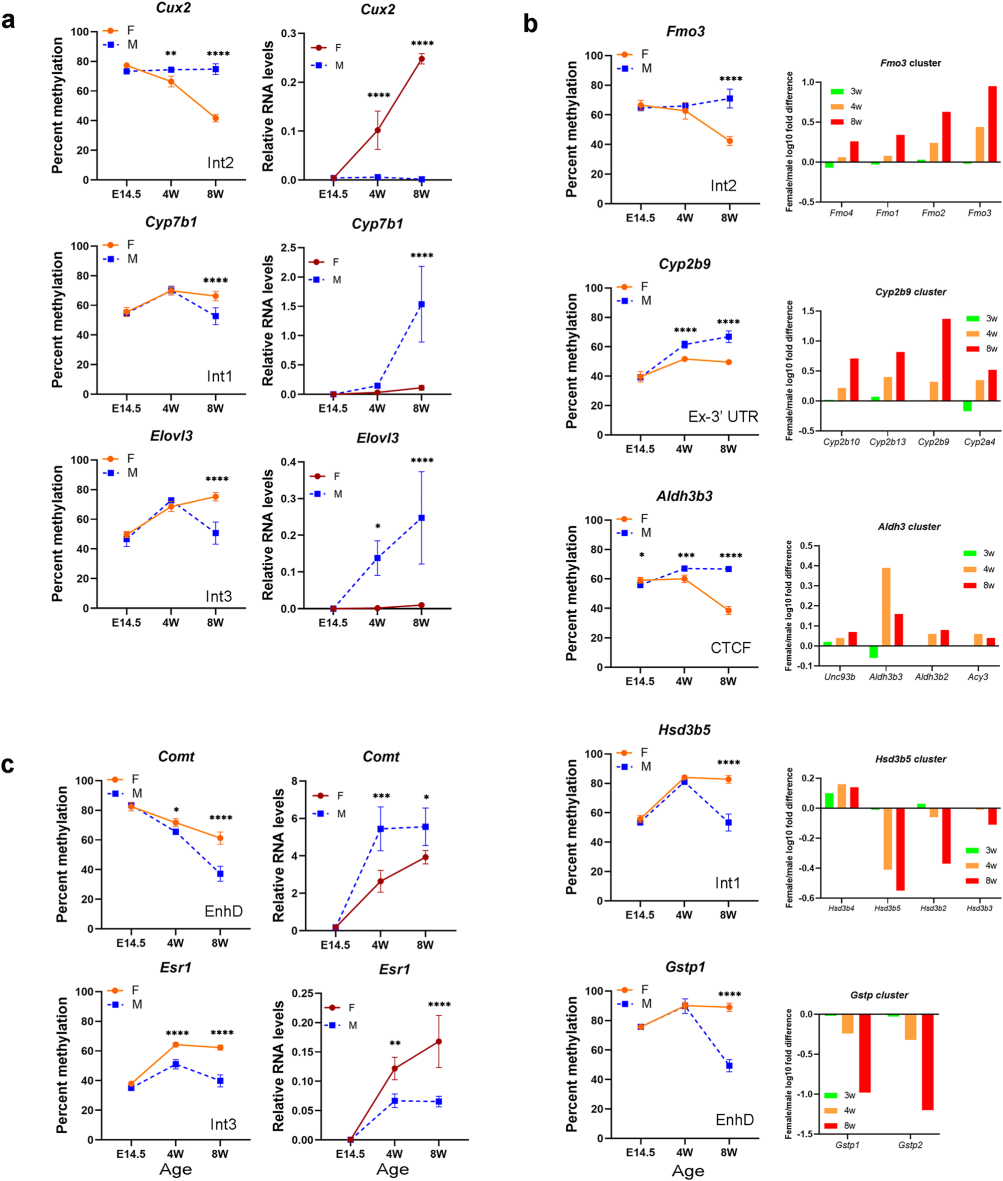
DNA methylation of sDMRs and proximal sDEGs expression during development. (**a**) Methylation levels of sDMRs and expression levels of proximal sDEGs for sDMRs associated with a single sDEG (4–6 females and 4–6 males at E14.5, 7 females and 4 males at 4 weeks, and 4 females and 6 males at 8 weeks of age); (**b**) Left panel, methylation levels of sDMRs with several proximal sDEGs**.** Right panel, log10 fold difference in gene expression between females and males from dataset GSE34782^9^. (**c**) Methylation levels of sDMRs and expression levels of proximal genes that did not reach the threshold for sDEG. Location of the sDMR with respect to genomic features is noted as: Int—intron; Ex—exon, EnhD—distal enhancer; CTCF—CTCF-bound region. Error bars show standard deviation. Statistically significant differences between sexes are shown with asterisks *P < 0.05, **P < 0.01, ***P < 0.001; ****P < 0.0001 (two-way ANOVA followed by multiple testing with Sidak’s correction).

Here, we focused on autosomal sex-phenotype dependent sDMRs, i.e. sDMRs whose methylation levels in phenotypic females, XX or XY, were different from methylation levels in phenotypic XY males^6^. We chose a targeted and sDMR-centered approach and examined three types of sDMRs, those that were located within gene bodies or promoter regions of single sDEGs; those associated with more than one sDEG located within 100 kb of the sDMR; and those that were not associated with sDEGs. Using our previously published WGBS and RNA-seq data^6^, we selected five regions that contained two or more sDEGs as well as sDMRs and included genes with a well-documented sex bias in expression and validated sex bias in methylation (Table S1). Regions with female-biased sDMRs and sDEGs were the *Cyp2* cluster on chromosome 7 containing 8 sDEGs; the *Fmo* cluster on chromosome 1 containing 3 sDEGs and 3 genes with sex bias that did not reach the threshold for DEG inclusion (log2FC ≥ 1.5, *P* < 0.05); and the *Aldh3* cluster on chromosome 19 containing 2 sDEGs (Figs. 1 and S1). Regions with male-biased sDMRs and sDEGs were the *Gstp* cluster on chromosome 19 containing 2 sDEGs and the *Hsd3b* cluster on chromosome 3 containing 2 sDEGs (Figs. 1 and S1). We also tested three sDMRs that were associated with only one proximal sDEG and located within the sDEG promoter region or gene body: female-biased *Cux2* and male-biased *Cyp7b1* and *Elovl3* (Figs. 1 and S2). Finally, two sDMRs located within gene bodies, but with no sDMR-proximal genes that reached the threshold for sDEG inclusion (log2FC ≥ 1.5, *P* < 0.05) were selected: the male-biased *Comt* and *Esr1* sDMRs (Figs. 1 and S2).

To establish the developmental dynamics of sex bias in DNA methylation and expression for the selected regions, we compared DNA methylation levels in female and male livers at three different ages, representing fetal (E14.5), prepubertal (4 weeks), and adult (8 weeks) life, using pyrosequencing methylation assays. DMRs located within the promoters of X-linked gene X inactive specific transcript (*Xist*) and imprinted gene small nuclear ribonucleoprotein N (*Snrpn*) were tested as controls (Fig. S3a). Expression levels of sDMR-proximal sDEGs, *Cux2*, *Cyp7b1*, *Elovl3*, *Comt*, *Esr1*, *Cyp2b9*, *Cyp2b13*, *Fmo3, Fmo2*, and *Aldh3b3*, were assayed in the livers of the same mice using qPCR (Figs. 1 and S3). Two genes with high expression in fetal liver, imprinted gene *H19* and the SRY (sex determining region Y)-box 6 gene (*Sox6*), were used as controls for qPCR to confirm the accuracy of expression profiles (Fig. S3b). For the other sDMR-proximal sDEGs or non-sex-biased neighboring genes, ratios between expression levels in female and male livers at 3-, 4- and 8-weeks of age were extracted from the publicly available dataset GSE34782^9^.

Statistically significant sex-bias in DNA methylation was detected at the same ages as bias in expression at male-biased *Cyp7b1*, *Comt*, *Esr1,* and *Gstp1* and female-biased *Cyp2b9, Aldh3b3,* and *Cux2* sDMRs (Figs. 1 and S3c). For the male-biased *Elovl3* and *Hsd3b5* and female-biased *Fmo3* sDMRs, sex bias in methylation was not detected at 4 weeks, while sex bias in expression of proximal sDEGs was already evident at this age (Figs. 1a, b and S3c). Sex bias in methylation at the *Comt* sDMR increased between 4 and 8 weeks while sex bias in expression was unchanged (Fig. 1c). For the *Cyp2b, Fmo,* and *Gstp* clusters, sDEGs located within the same cluster had similar developmental dynamics (Figs. 1b and S3c).

Hence, in our panel of 10 sDMRs, we find dynamic changes of DNA methylation with age (Fig. 1). At 5 of the 6 male-biased sDMRs, methylation levels did not change between 4 and 8 weeks in females but were reduced in males, whereas at 3 of the 4 female-biased sDMRs, methylation levels did not change between 4 and 8 weeks in males but were reduced in females. Therefore, in 8 of the 10 sDMRs, the dynamics of methylation is consistent with demethylation in one sex, but not the other. The profile of the *Cyp2b9* sDMR methylation is suggestive of reduced gain of methylation with age in females, whereas the *Comt* sDMR shows a trend of loss of methylation with age in both sexes, but males lose methylation faster. In all tested sDMRs, sex bias in methylation did not precede sex bias in expression. In three sDMRs, one female-biased and two male-biased, sex bias in methylation appeared after the sex bias in expression was already established.

### Presence of both, ovary and testis, delays sDMR demethylation in XY hermaphrodites

True hermaphrodites carry both testes and ovaries and represent a unique model where the same mouse is exposed to testosterone and estrogen during development^16^. The Tirano Y chromosome (Y^*TIR*^) harbors a sex-determining region Y (*Sry*) gene variant that encodes an SRY protein that is shorter than the C57BL/6J variant. On the C57BL/6J genetic background, the Tirano variant fails to efficiently upregulate its target gene SRY (sex determining region Y)-box 9 (*Sox9*), which is necessary for initiating testicular differentiation^17–21^. This causes variability in gonadal sex differentiation: XY^*TIR*^ mice may develop bilateral ovaries with female sex phenotype, bilateral testes with male phenotype, or a unilateral testis and a contralateral ovary (or streak gonad) with an intersex phenotype^16,22,23^. Hermaphrodites also have 1/5 to 1/10 of the testosterone levels observed in adult XY^*TIR*^ males^16,24^. Moreover, sex-reversed females and hermaphrodites lose most of the ovarian follicles by 8 weeks of age and fail to establish estrus cyclicity^23^.

**Figure 2:**
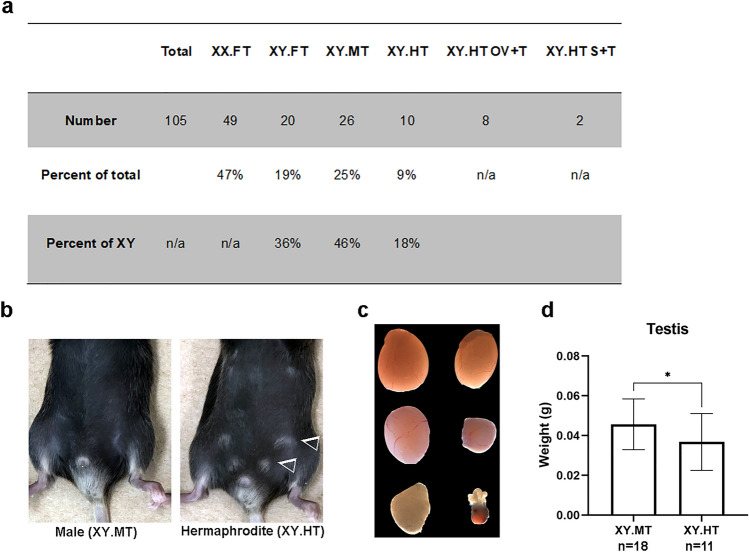
Characteristics of true hermaphrodites from the B6.Y^*TIR*^ cross. (**a**) Distribution of genotypes and sex phenotypes among the offspring of XY^*TIR*^ males. (**b**–**d**) Male (XY.MT) and hermaphrodite (XY.HT) phenotypes. (**b**) Different anogenital distance and mammary gland development in males and hermaphrodites (arrowheads point to mammary glands); (**c**) Males show variable testes weight with either two testes of similar weight (top row) or one testis larger than the other (middle row). Hermaphrodites have unilateral small testis and contralateral ovary (XY.HT OV + T) (bottom row) or a streak gonad (XY.HT S + T) (not shown); (**d**) Testis weight in males and hermaphrodites. Error bars show standard deviation. Asterisk indicates significant difference *P < 0.05 (one-tailed t-test).

In the B6.Y^*TIR*^ crosses that were used for this study, XY females accounted for about 40% of XY progeny, whereas slightly less than 20% were hermaphrodites (Fig. 2a). Most hermaphrodites had male-like external genitalia with shorter anogenital distance compared to males and often showed signs of mammary gland development (Fig. 2b). Hermaphrodites had smaller testes than males (*P* < 0.05, one-tailed t-test) (Fig. 2c, d). It is also worth noting that testis weight was highly variable in XY^*TIR*^ males (Fig. 2c, d). Ovarian follicles were observed in only one of the 8-week old hermaphrodites.

**Figure 3:**
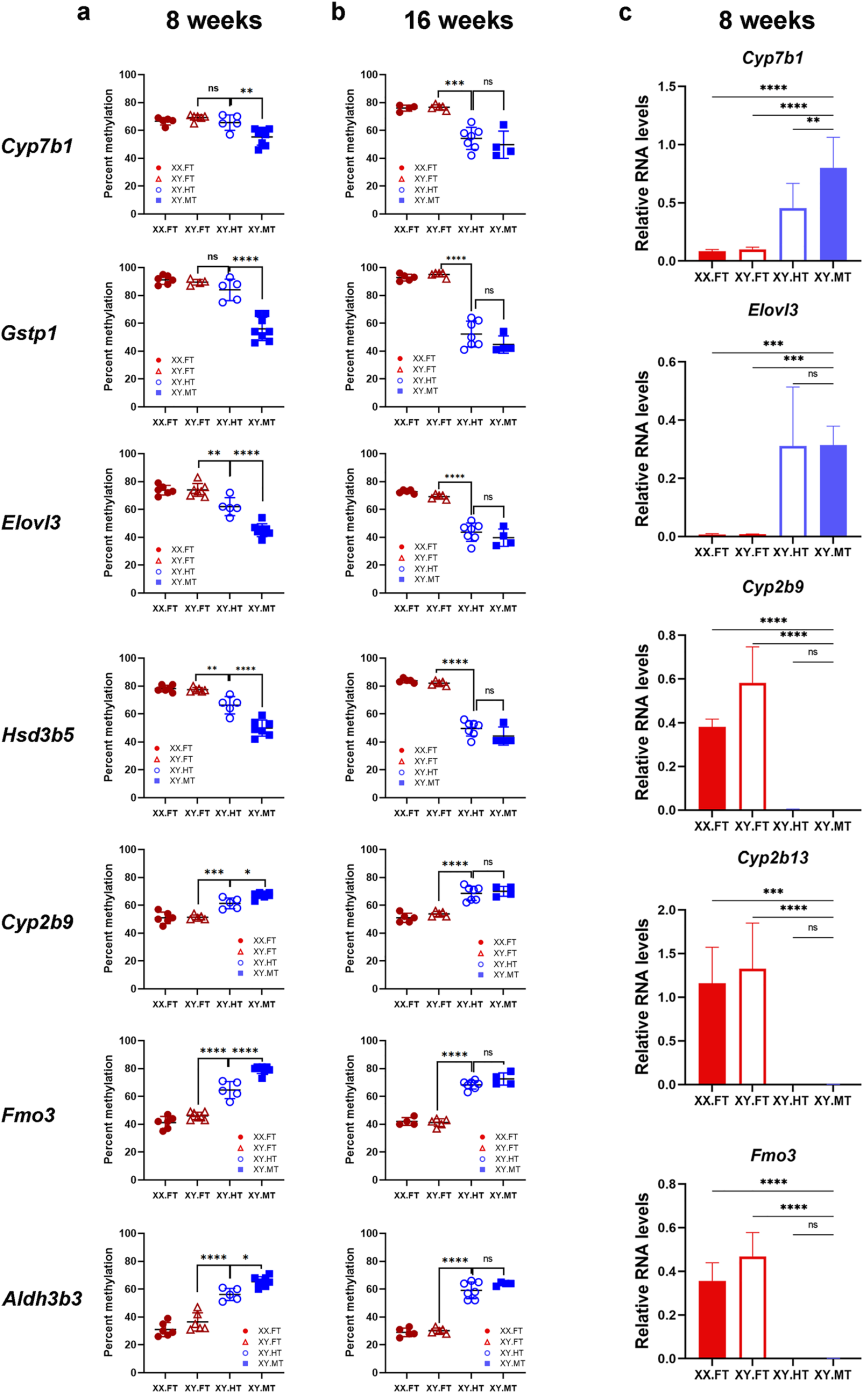
Dynamics of DNA methylation and proximal sDEG expression in hermaphrodites. (**a**–**b**) DNA methylation levels in true hermaphrodites (XY.HT, n8w = 5, n16w = 7) in comparison to sex-reversed females (XY.FT, n8w = 6, n16w = 5) and males (XY.MT, n8w = 8, n16w = 4) at 8 (**a**) and 16 (**b**) weeks of age. (**c**) Relative expression levels of sDEGs normalized to *Rpl19* in 8-week old mice from the B6.Y^*TIR*^ cross (5 XX.FT, 6 XY.FT, 5 XY.HT, and 8 XY.MT livers). Error bars show standard deviation. Statistically significant differences are shown with asterisks *P < 0.05, **P < 0.01, ***P < 0.001, ****P < 0.0001, ns: non-significant (one-way ANOVA, followed by multiple testing with Tuckey’s correction). All tested sDMRs were selected based on their previously validated significant differences between females (XX.FT or XY.FT) and males (XY.MT)^6^, hence these differences are not indicated by asterisks.

We compared methylation levels in 8- and 16-week old XY^*TIR*^ mice with the three different sex phenotypes (females (XY.FT), true hermaphrodites (XY.HT), and males (XY.MT)) at seven sDMRs (Fig. 3a, b). At male-biased sDMRs, 8-week old hermaphrodites had either female-type (*Cyp7b1* and *Gstp1* sDMRs) or intermediate (*Elovl3* and *Hsd3b5* sDMRs) methylation levels (Fig. 3a). At the 3 female-biased sDMRs, hermaphrodites had intermediate methylation levels. However, at 16 weeks, the methylation levels at all 7 sDMRs were not significantly different between hermaphrodites and males (Fig. 3b). Hence, our data suggest that adult hermaphrodites may have female-like or intermediate methylation patterns but subsequently develop male-like methylation patterns at sDMRs.

We next asked if the sex-biased expression of sDMR-proximal genes showed the same delay and assayed the expression of *Cyp7b1, Elovl3, Cyp2b9*, *Cyp2b13*, and *Fmo3* genes in 8-week old XY.HT mice and their littermates (Fig. 3c). XY.HT mice had intermediate levels of *Cyp7b1* compared to XY.FT and XY.MT mice, whereas the expression levels of female-biased genes *Cyp2b9*, *Cyp2b13*, and *Fmo3,* and the male-biased *Elovl3* were not different between hermaphrodites and males (Fig. 3c). These data suggest that male-type expression patterns are established before male-type methylation patterns in XY.HT animals.

XY.FT, XY.MT, and XY.HT mice share the same sex-chromosome complement and genetic background. The fundamental difference between the XY.FT and XY.HT is that XY.FT have two ovaries, whereas XY.HT have an ovary and a testis. Hence, methylation differences between these two groups reflect the influence of the testis. XY.MT are males and have bilateral testes. Hence, differences in methylation between XY.MT and XY.HT must be due to the presence of the ovary. Thus, our data demonstrate that presence of both ovary and testis influences the dynamics of DNA methylation at the sDMRs.

### Developmental profiles of sex-biased expression of liver TFs

**Figure 4:**
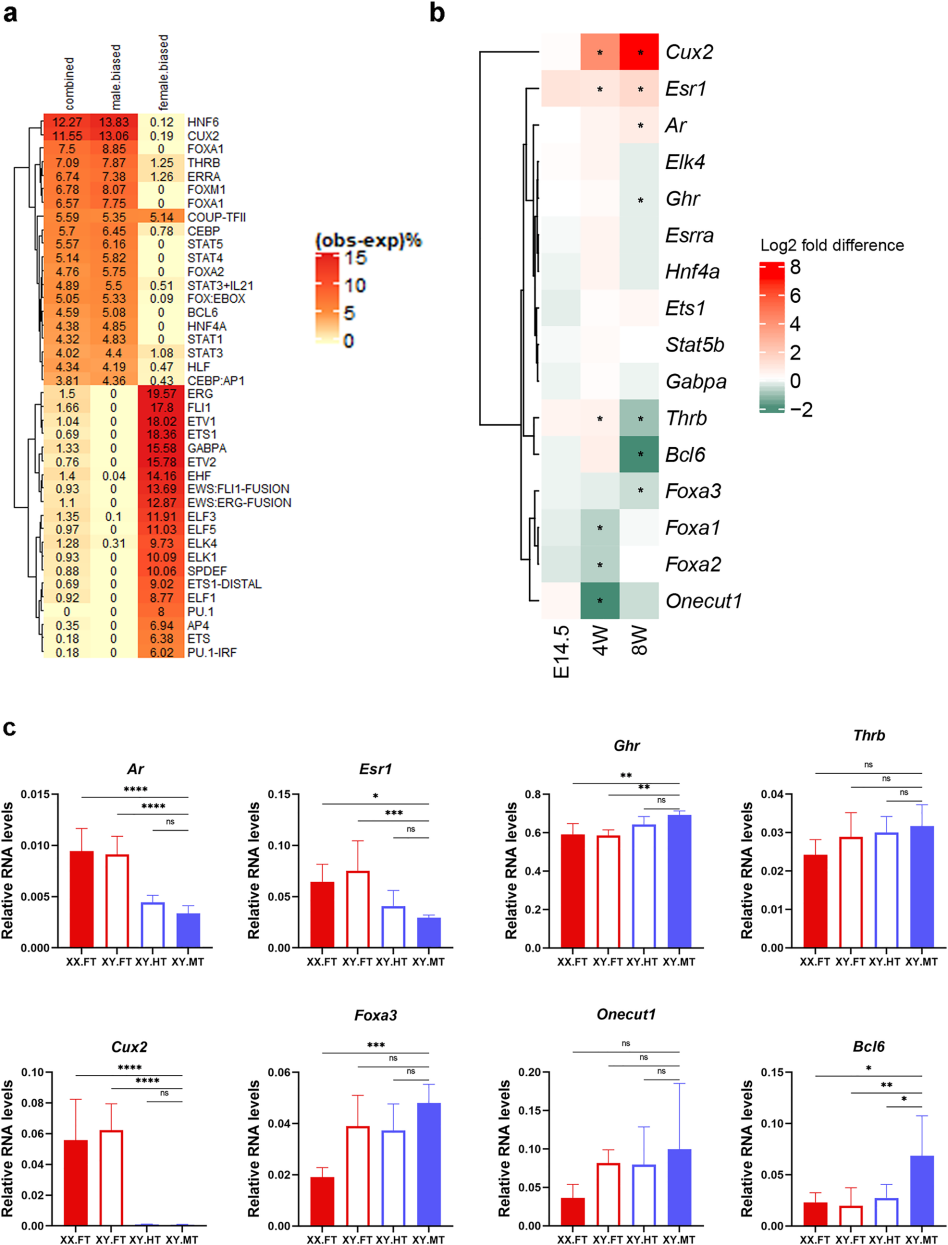
Transcription factors associated with sDMRs. (**a**) HOMER motif analysis results for autosomal sDMRs associated with sex phenotype identified in the XY.F versus XY.M comparison (n = 3847)^6^, as well as male-biased (n = 3414) and female-biased (n = 433) sDMRs, separately. Number, reflected as color intensity, represents the motif enrichment level, i.e. the difference between the observed frequency (in %) in sDMRs less the frequency in randomly generated background regions. Only motifs with relative frequencies observed in sDMR higher than expected relative frequencies in randomly generated background regions were shown. (**b**) Developmental expression profiles of genes encoding TFs. Heatmap shows the log2 of fold differences between C57BL/6J females and males (ratio female over male expression) at different ages. Asterisks indicate statistically significant sex difference. (**c**) Relative expression levels of genes encoding TFs normalized to *Rpl19* in XY hermaphrodites and their littermates at 8 weeks of age. Error bars show standard deviation. Statistically significant differences are shown with asterisks *P < 0.05, **P < 0.01, ***P < 0.001, ****P < 0.0001, ns: non-significant (one-way ANOVA, followed by multiple testing with Tuckey’s correction for comparisons to the XY.MT group).

Sex-biased methylation of autosomal sDMRs may result from sex bias in the levels of TFs that bind the sDMRs. Hence, to prioritize candidate TFs, we analyzed motif enrichment in all sex-phenotype dependent sDMRs (previously identified in the comparison between sex-reversed XY females and XY males^6^). Male- and female-biased sDMRs were also analyzed separately (Fig. 4a). DNA-binding motifs for TFs with well-established roles in sex-biased expression (e.g. CUX2, HNF6 (ONECUT1), STAT5, BCL6, FOXA1/FOXA2) were enriched at male-biased sDMRs, whereas binding motifs for the ETS-family of TFs were enriched at female-biased sDMRs (Fig. 4a). Although binding motifs for androgen and estrogen receptors were not among the top enriched motifs, these receptors are essential for testosterone and estradiol genomic actions and therefore logical candidates for controlling sex bias in DNA methylation.

We next established the expression profiles of genes encoding TFs that have their binding motifs enriched at male-biased (*Onecut1*, *Cux2*, *Foxa1*, *Thrb*, *Esrra*, *Foxa2*, *Bcl6, Hnf4a, Stat5b*)*,* or female-biased (*Ets1*, *Gabpa*, *Elk4*) sDMRs and are highly expressed in liver using qPCR (Figs. 4b and S4). We also included *Ar*, *Esr1*, and *Foxa3*, another factor from the FOXA family, in the panel. However, estrogen receptor beta (*Esr2*), which is expressed at extremely low levels in the mouse liver was not analyzed. We also tested the liver expression levels of the growth hormone receptor gene (*Ghr*) that is critical for GH-signaling, albeit not a transcription factor.

Sex bias was detected in the expression of 10 of the 16 tested genes at different ages (Figs. 4b and S4). *Foxa1*, *Foxa2*, and *Onecut1* showed significant sex bias in the livers of 4-week old mice, but not in fetal or adult livers. *Cux2*, *Esr1*, and *Thrb* showed sex bias in 4- and 8-week old mice. *Ar*, *Ghr*, *Foxa3*, and *Bcl6* showed significant sex bias in 8-week old, but not in fetuses or 4-week old mice. Expression of 13 genes increased with age, however two genes, *Foxa2* in males and *Bcl6* in females, showed a downward trend between 4 and 8 weeks of age (Fig. S4).

We hypothesized that the factor that was responsible for the delay in demethylation of male-biased sDMRs in hermaphrodites, would have a female-type expression levels in 8-week old hermaphrodites (XY.HT). Expression levels of eight TFs that showed sex bias were tested in 8-week old XY.HT, XY.FT, XY.MT, and XX.FT livers (Fig. 4c). *Bcl6* expression levels in hermaphrodites were lower compared to males, but not different from females. In contrast, the 7 other genes showed male-like levels of expression in hermaphrodites or no difference between groups. These data warranted an investigation of the role of *Bcl6* in demethylation of sDMRs.

### Loss of BCL6 prevents demethylation of both male-biased and female-biased sDMRs

**Figure 5:**
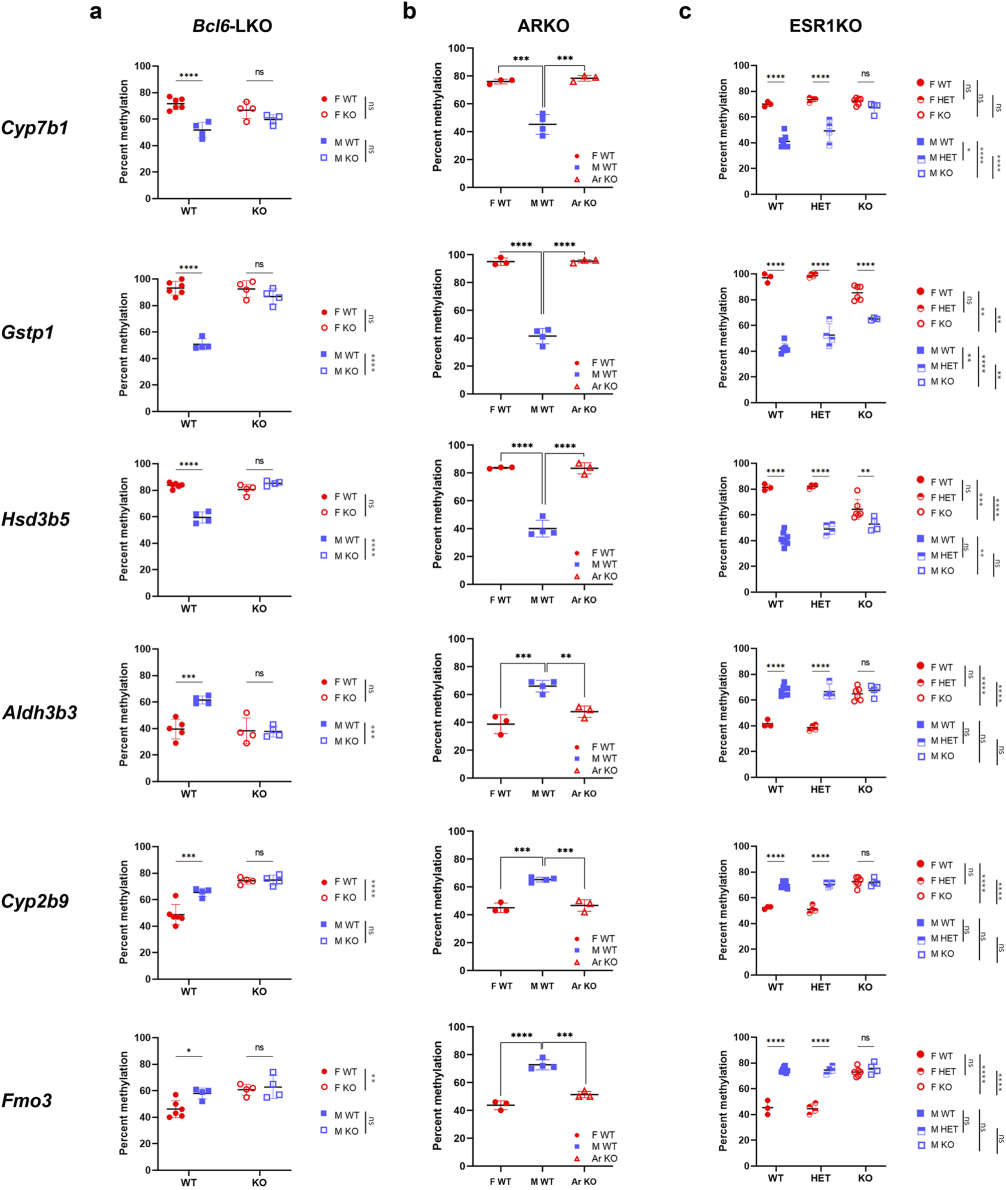
BCL6, AR, and ESR1 influence DNA methylation at sDMRs. (**a**) Methylation levels in male and female *Bcl6*-LKO (4 females, 4 males) and controls *Bcl6*-flox mice (6 females, 4 males); (**b**) Methylation levels in ARKO mice (with genetic and gonadal male sex and genital female sex) and control male and female littermates (3 females, 4 males, and 3 ARKO mice); (**c**) Methylation levels in wild type controls, heterozygous and homozygous ESR1KO mice (WT: 3 females, 7 males; HET: 4 females, 4 males, KO: 6 females, 4 males). Error bars show standard deviation. Statistically significant differences are shown with asterisks *P < 0.05, **P < 0.01, ***P < 0.001, ****P < 0.0001, ns: non-significant [two-way ANOVA, followed by multiple testing with Sidak’s correction (**a** and **c**); one-way ANOVA, followed by multiple testing with Tuckey’s correction (**b**)].

Several lines of evidence suggest that BCL6 contributes to sex-biased gene regulation, potentially as a repressor of female-biased genes in the male liver^14,25^. To determine if BCL6 influenced DNA methylation, we examined sDMR methylation levels in the livers of mice carrying a hepatocyte-specific deletion of *Bcl6* (*Bcl6*-LKO) and their littermates that carry a floxed *Bcl6* allele (*Bcl6*^*flox/flox*^). The *Bcl6*-LKO males had higher methylation levels at the male-biased *Cyp7b1, Gstp1*, and *Hsd3b5* sDMRs, whereas *Bcl6*-LKO females had higher methylation levels at the female-biased *Cyp2b9* and *Fmo3* sDMRs compared to controls. The *Aldh3b3* sDMR was the only region with lower methylation in the *Bcl6*-LKO compared to wild type males (Fig. 5a).

Thus, loss of BCL6 in hepatocytes led to loss of sex differences in methylation levels, as predicted based on previously observed loss of sex-biased expression in the livers of these mice^14^. Given the well-documented role of BCL6 as a male-specific transcriptional repressor^12,14,26^, one would expect that its loss would be associated with lower methylation levels at female-biased sDMRs in males. Contrary to expectations, deletion of *Bcl6* prevented demethylation in 5 of the 6 tested sDMRs, both female- and male-biased. Only the female-biased *Aldh3b3* sDMR showed reduced methylation in males. This suggests that in the mouse liver, BCL6 functions are not limited to transcriptional repression and may include a role in demethylation of sDMRs.

### AR and ESR1 influence methylation levels at sex-phenotype dependent sDMRs

To better understand the roles of androgen and estrogen receptors in sex-biased methylation, we tested the methylation levels of sDMRs in adult mice that carry mutations in *Ar* or *Esr1*. The ARKO mice carry a constitutive deletion of the exon 3 of *Ar* that abolishes the DNA-binding activity of the protein but preserves its other functions^27^. XY ARKO hemizygous mice carry intraabdominal small testes but develop female external genitalia due to androgen insensitivity^27^. Hence, their genetic and gonadal sex is male, but the phenotypic sex is female. Liver DNA methylation of the XY ARKO mice was analyzed using a panel of 6 sDMRs: male -biased *Cyp7b1*, *Gstp1*, and *Hsd3b5*, and female-biased *Aldh3b3*, *Cyp2b9*, and *Fmo3.* XY ARKO mice showed female-type methylation levels at all 6 tested sDMRs (Fig. 5b).

The ESR1KO mice carry a deletion of exon 3, which abolishes ESR1 production and causes infertility and elevated testosterone levels in both males and females^28^. Homozygous mutants have dramatically reduced levels of *Esr1* RNA in their livers (Fig. S5). We analyzed methylation levels in the ESR1KO mice using the same panel of 6 sDMRs (Fig. 5c). Loss of *Esr1* led to loss of sex bias in methylation at the female-biased sDMRs with the ESR1KO females displaying high methylation levels similar to those observed in males. At male-biased sDMRs *Cyp7b1*, *Gstp1*, and *Hsd3b5,* the sex bias in methylation was either lost or reduced, with ESR1KO females having lower methylation levels and males having higher methylation levels compared to wild type mice (Fig. 5c).

In summary, all three mutations are associated with hypermethylation of male-biased sDMRs in genetic males. However, their impacts on female-biased sDMR in males and both male-and female-biased sDMRs in females vary. This suggests distinct roles for these factors in the establishment or maintenance of sex-biased DNA methylation. These roles may result from direct and liver-specific functions or may be mediated through the gonadal-pituitary axis as suggested by other studies (reviewed in^10^).

### ESR1 and BCL6 have different roles in sex-biased gene expression

**Figure 6:**
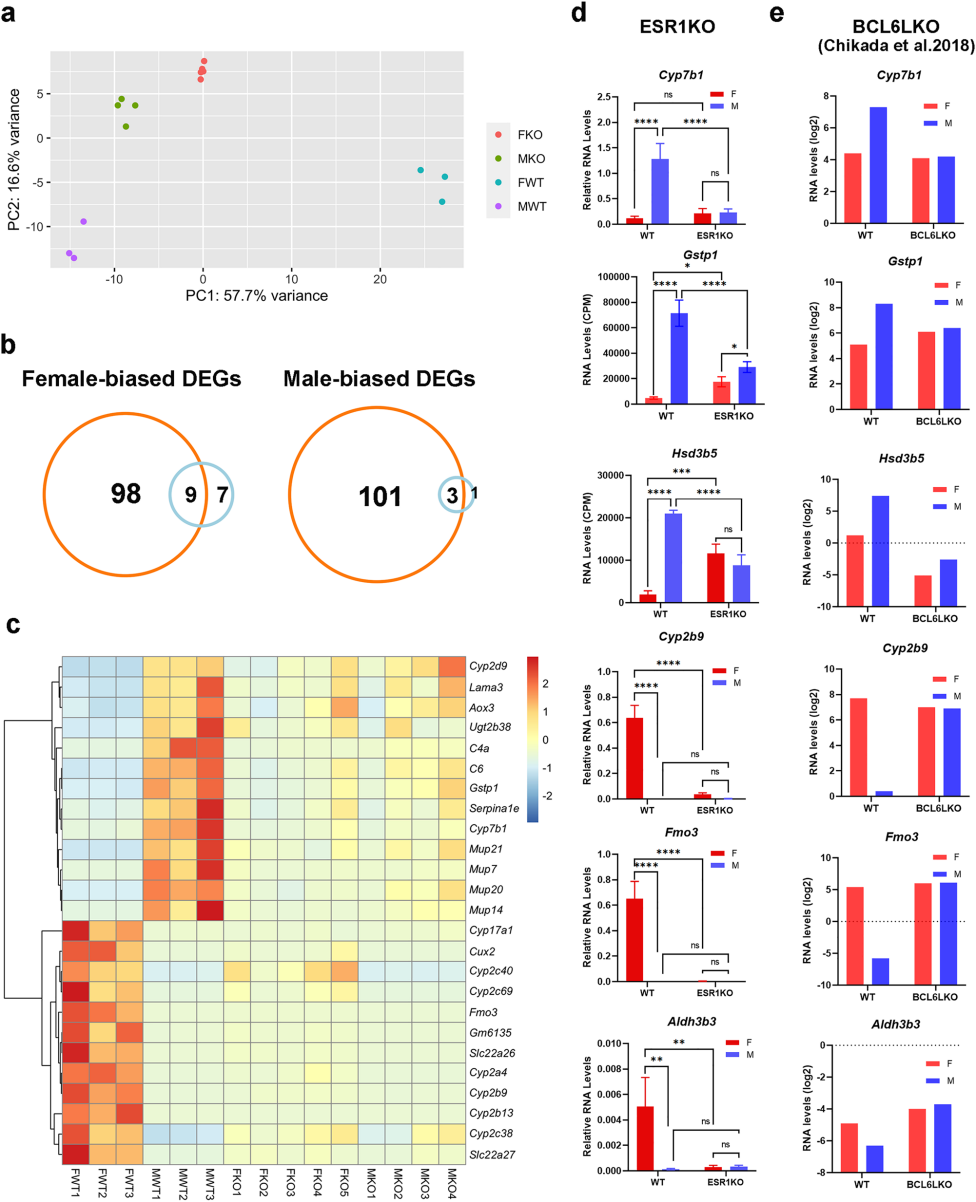
Ablation of ESR1 is associated with loss of sex bias in gene expression. (**a**) Principal component analysis (PCA) for RNA-seq data from ESR1KO and control mouse livers shows that samples cluster by genotype and sex, when comparing the first two principal components. (**b**) Overlapping DEGs between WT (orange) compared to the ESR1KO (blue): female-biased DEGs (left panel) and male-biased DEGs (right panel) are shown separately. (**c**) Heatmap shows the top 22 significant sDEGs as well as *Cyp2b9, Cyp2b13*, and *Fmo3* in WT and ESR1KO mice. (**d**) Relative expression levels of sDEGs in ESR1KO mice determined by RT-qPCR and normalized to *Rpl19* (WT: 3 females and 3 males, ESR1KO: 4 females and 4 males) or retrieved from RNA-seq data (*Gstp1, Hsd3b5*). Error bars show standard deviation. Statistically significant differences between sexes are shown with asterisks *P < 0.05, **P < 0.01, ***P < 0.001, ****P < 0.0001, ns: non-significant (two-way ANOVA followed by multiple testing with Sidak’s correction). (**e**) Expression levels of sDEGs in *Bcl6*-LKO mice (data from GSE89091 and GSE107435 displayed as log2 of normalized signal intensity).

To better understand the relationship between sex-biased methylation and sex-biased expression, we next examined the expression profiles of sex-biased genes in two mutant strains, ESR1KO and *Bcl6*-LKO. The liver transcriptomes of ESR1KO and control mice were analyzed using RNA-seq (data have been deposited to the NCBI GEO repository, GSE174535) (Fig. 6a–d). For *Bcl6*-LKO mice, liver expression data were extracted from previously published GSE89091 and GSE107435 microarray datasets^14^ (Fig. 6e).

Principal component analysis (PCA) of the expression data showed that the liver transcriptomes of ESR1KO females and males form distinct clusters and tend to be closer to those of wild type males than wild type females (Fig. 6a). Mutant mice lose sex bias in the expression of 94% of sDEGs detected in wild type mice (Fig. 6b), which is largely associated with downregulation of male-biased genes in males and female-biased genes in females (Fig. 6c, d). These trends were confirmed in our panel of sDEGs by RT-qPCR assays: expression of female-biased genes *Cyp2b9, Fmo3,* and *Aldh3b3* was lower in ESR1KO females compared to wild type females, and expression of male-biased genes *Cyp7b1*, *Hsd3b5,* and *Gstp1* was lower in ESR1KO males compared to controls. In the ESR1KO females, *Gstp1* and *Hsd3b5* were upregulated compared to wild type females (Fig. 6c, d). Thus, across the 6 sDMRs and 6 sDEGs in our panel, methylation mirrored expression differences between ESR1KO and WT mice.

Expression of male-biased genes in the *Bcl6*-LKO males was lower than in controls, consistent with higher methylation at associated sDMRs and similar to the trends observed in ESR1KO males. In the *Bcl6*-LKO females, no differences were found for the male-biased sDEGs *Cyp7b1* and *Gstp1*, which was consistent with lack of changes in methylation at the associated male-biased sDMRs. Expression of female-biased genes *Cyp2b9* and *Fmo3* in the *Bcl6*-LKO females was not different from controls. This was in contrast to hypermethylation of *Cyp2b9* and *Fmo3* sDMRs. For *Aldh3b3*, no differences in expression nor sDMR methylation were found. Expression of female-biased genes was higher in the *Bcl6*-LKO males compared to controls, also in contrast to trends in methylation. Thus, ablation of BCL6 exposes a dissociation of sDMR methylation from expression of proximal sDEGs, which is somewhat reminiscent of the data from 8-week old hermaphrodites.

### Co-localization of sDMRs with ESR1, AR, and BCL6 enriched regions

In principle, there are at least three possible but not mutually exclusive mechanisms by which sex hormone receptors may influence DNA methylation. Firstly, they may directly bind sDMRs or their adjacent regions. Secondly, they may bind distant enhancers or silencers, thereby influencing gene expression, and consequently DNA methylation through long-range chromatin interactions^29^. Thirdly, their effects may be mediated through other signaling pathways with the downstream effectors of these pathways modifying methylation, gene expression, and chromatin states (reviewed in^10^).

**Figure 7:**
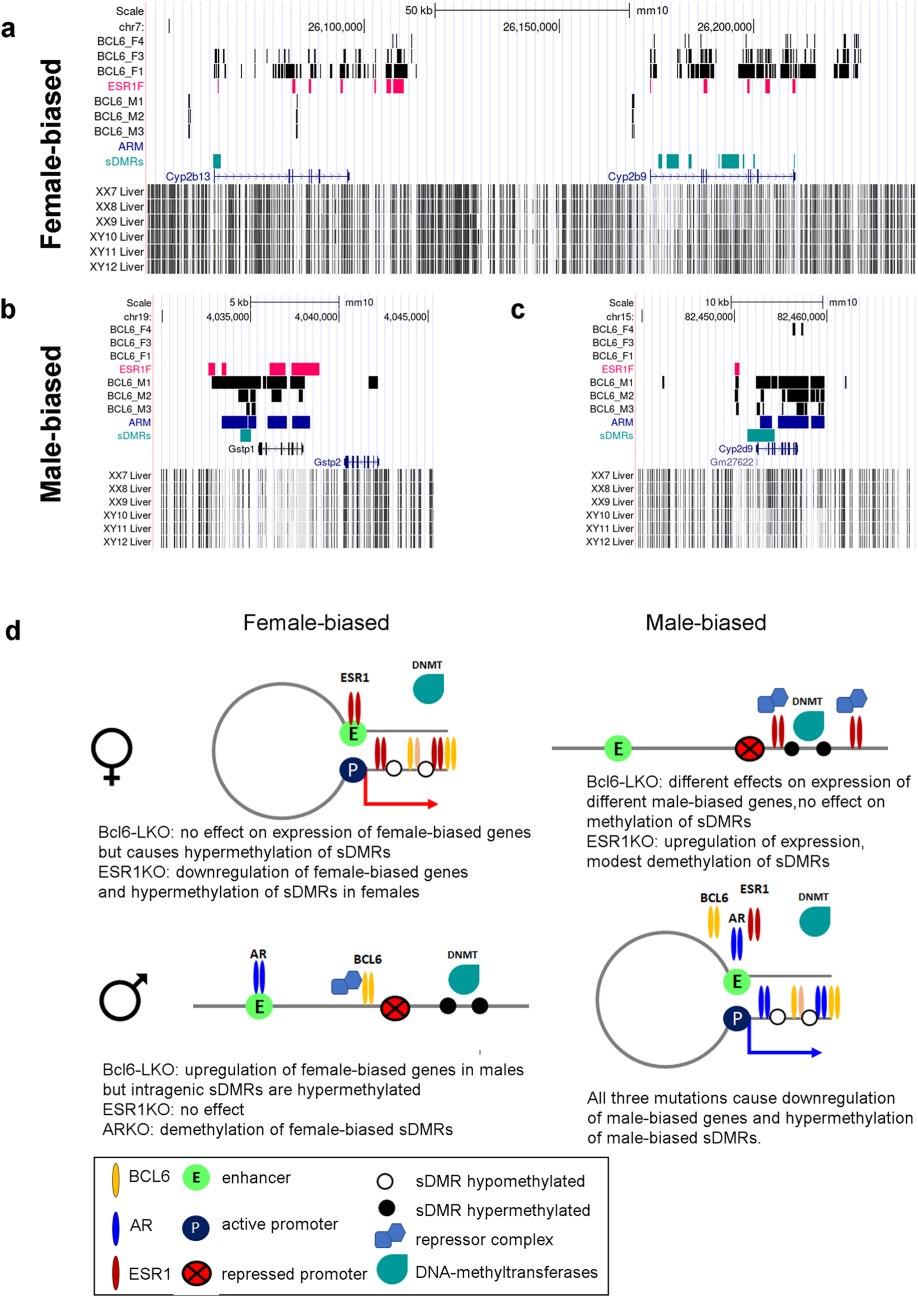
Co-localization of AR, ESR1, and BCL6 enrichment with sDMRs and proposed working hypothesis. (**a**–**c**) Examples of overlapping sDMRs, sDEGs, BCL6, AR, or ESR1 enrichment; at female-biased sDMRs (**a**); and male-biased sDMRs (**b** and **c**). All tracks are shown in the context of the UCSC genome browser (mm10). BCL6-F1, BCL6-F3, and BCL6-F4 tracks show ChIP-seq peaks for BCL6 in the female liver (replicates 1, 3, and 4, respectively). BCL6-M1, BCL6-M2, and BCL6-M3 show ChIP-seq peaks for BCL6 in the male liver (replicates 1, 2, and 3, respectively)^12^. The ESR1F track shows ChIP-seq peaks for ESR1 in the female liver, and the ARM track shows ChIP-seq peaks for AR in the male liver^30^. The “sDMRs” track shows positions of sDMRs from the XX.F versus XY.M comparison based on WGBS data from^6^. The XX7, XX8, XX9, XY10, XY11, and XY12 liver tracks show positions of CG sites and the intensity of the tick reflects methylation level (WGBS data from^6^), in females (XX) and males (XY). (**d**) Schematic representation of the working hypothesis.

If AR, ESR1, or BCL6 influenced DNA methylation through binding to sDMRs or in close proximity to sDMRs, sDMRs would co-localize with AR, ESR1, or BCL6-bound chromatin. To test this prediction, we examined the co-localization of sex-phenotype dependent sDMRs with AR-, ESR1-, or BCL6-enriched regions, using our previously published WGBS data and publicly available ChIP-seq data for mouse liver (AR/ESR1: GSE32244, BCL6: GSE31578). Significance of the results was tested by comparing the percent overlap with sDMRs to 500 permutations for each of the ChIP datasets. Co-localization analysis showed sex bias in the co-localization of sDMRs and AR or BCL6 ChIP peaks in male liver with significant overlap (Table 1). We next examined the distribution of distances between sDMRs and closest AR, ESR1, or BCL6 enrichment sites (ChIP-seq peaks) and observed a tendency of male-biased sDMRs to localize closer to BCL6-enriched sites in both male and female livers, AR-sites in male livers and ESR1-sites in female livers, compared to random permutation controls (Fig. S6 and Table 1). Female-biased sDMRs showed a tendency to be closer to ESR1- or BCL6-sites in female livers compared to random controls (Fig. S6 and Table 1). These data suggest that a substantial proportion of sDMRs tend to reside in the vicinity of BCL6, AR, or ESR1-bound regions. Close inspection of some of the sDMRs and sDEGs that co-localize with BCL6 reveals BCL6 enrichment that overlaps with the sDMR or the sDEG gene body in a sex-dependent fashion, and either ESR1-bound regions in the female liver or AR-bound regions in the male liver (Fig. 7a–c). Importantly, BCL6 is enriched in the sex with active expression of the given gene, suggesting a role that is different from transcriptional repression and may include interaction with ESR1 or AR.

**Table 1:** Co-localization of autosomal sDMRs with BCL6, AR, or ESR1-enriched regions.

	Sex-phenotype dependent sDMRs	Female liver				Male liver			
		Co-localized with BCL6-ChIP enriched regions** (% sDMRs)		Co-localized with ESR1-ChIP enriched regions*** (% sDMRs)		Co-localized with BCL6-ChIP enriched regions** (% sDMRs)		Co-localized with AR-ChIP enriched regions**** (% sDMRs)	
		Direct Overlap	Within 10 kb	Direct Overlap	10 kb	Direct Overlap	10 kb	Direct Overlap	10 kb
Female-biased sDMRs	433	12 (2.8)*	133 (30.7)*	26 (6.0)*	153 (35.3)*	4 (0.9)	100 (23.1)*	1 (0.2)	46 (10.6)*
Male-biased sDMRs	3414	81 (2.4)*	932 (27.3)*	255 (7.5)*	1449 (42.4)*	413 (12.1)*	1573 (46.1)*	87 (2.5)*	721 (21.1)*
Total sDMRs	3847	103 (2.7)	1065 (27.7)	295 (7.7)	1602 (41.6)	477 (12.4)	1673 (43.5)	97 (2.5)	767 (19.9)
Total number of autosomal ChIP peaks	n/a	22,851		31,235		20,994		7850	

*Statistical significance of co-localization of female-biased or male-biased sDMRs with TF-enrichment was determined by comparing the co-localization of 500 permutations with ChIP peaks. Asterisks indicate statistically significant results.

**BCL6-ChIP peaks in female and male livers extracted from GSE31578- GSM784032, GSM784028 replicates 3 and 2, respectively; ***ESR1-ChIP peaks in female liver extracted from GSE32244; ****AR-ChIP peaks in male liver extracted from GSE32244.

## Discussion

The main objective of this study was to better understand how sex-phenotype dependent methylation differences arise, delineate the distinct contributions of AR, ESR1, or BCL6 on sex-phenotype dependent DNA methylation and examine the relationship between sex-biased methylation and sex-biased expression in the mouse liver. We chose a targeted approach focusing on a panel consisting of both male- and female-biased autosomal sDMRs located within or close to genic regions, and with methylation levels depending on the sex phenotype, but not the sex-chromosome complement.

In the present study, we demonstrate that sex phenotype-dependent autosomal DNA methylation levels in the mouse liver depend on not one, but several factors, including AR, ESR1, and BCL6. We find that methylation levels at all 6 sDMRs from our test panel are modified by the global loss of either AR or ESR1, suggesting that both receptors contribute to the establishment or maintenance of sex bias in DNA methylation. Previous studies have focused on the more abundant male-biased sDMRs and suggested that testosterone signaling was required for the demethylation of male-biased sDMRs in males, whereas less was known about the contribution of estradiol signaling and the mechanisms driving the establishment of female-biased sDMRs^5,8,31,32^. By analogy to the reported impact of testosterone on demethylation of male-biased sDMRs, we expected that estradiol signaling would be involved in demethylation of female-biased sDMRs. Contrary to expectations, we find that ablation of the AR genomic function affects both DNA demethylation and gain/maintenance of DNA methylation. Furthermore, loss of ESR1 impacts methylation in both females and males across all 6 sDMRs in our panel. In males, it interferes with demethylation of the male-biased sDMRs that are also sensitive to the loss of AR. These data imply a wider than expected effect of these TFs and an interaction between androgen and estradiol signaling. The strongest evidence for interaction between the two pathways in shaping sex-specific DNA methylation patterns comes from the dynamics of DNA methylation in the livers of true hermaphrodites (Fig. 3). Here, we find that demethylation of the male-biased sDMRs is delayed until the age when ovarian function is lost.

Our data show that sex bias in DNA methylation appears around puberty, but is absent in fetal liver, in agreement with other reports ^8^. Given the exposure of fetuses to maternal estrogens during gestation and the importance of estrogen signaling for methylation in both sexes, as demonstrated here and in other studies, the inevitable conclusion is that fetal livers must be protected from the action of maternal estrogens. Moreover, in males, the fetal testes start producing testosterone around E13.5 (reviewed in^33^). The levels of testosterone surge at E16.5 and soon after birth before stabilizing at high levels after puberty^34,35^. Nevertheless, only a small subset of sDMRs are detected in prepubescent livers, as shown in our study and in^8^ (Fig. 1). Therefore, male fetal livers must be protected from the action of testosterone as well.

Alpha-fetoprotein (AFP), which is produced by the fetal liver but shows a sharp decline in expression between 10 and 14 days after birth^36^, is a plausible candidate factor for blocking estrogen action and thereby preventing demethylation of sDMRs in fetal livers. AFP effectively binds estrogens, but not testosterone; hence its decline after birth removes the barrier protecting the liver from the action of estradiol^37,38^. The sex-hormone binding globulin (SHBG) that binds testosterone and estrogens is another candidate protective agent^39,40^. Similar to AFP, its expression is high in fetal, but lost in the adult liver, which would explain the protection of fetal livers against maternal estrogens as well as testosterone produced by the fetal testes in males and the establishment of sex-biased expression and methylation postnatally after the drop in *Shbg* expression^41,42^. Finally, the genomic functions of sex hormones may be limited by the low availability of their receptors. Indeed, hepatic expression of *Ar* is low before puberty, however *Esr1* shows an earlier upregulation and high levels in 4 and 8-week old females (Fig. S4). Further studies of critical developmental time points in sex-biased methylation are necessary for better understanding of the mechanisms involved.

In liver and other tissues, sex bias in DNA methylation mirrors sex bias in gene expression^6,8^, leading to a question of which one is the cause, and which one is the consequence. The ensemble of our data suggests that at least for some loci sex bias in expression of sDMR-proximal genes precedes the establishment of sex bias in methylation. Indeed, 8-week old hermaphrodites show male-like expression levels of *Cyp2b9*, *Fmo3,* and *Elovl3*, whereas the methylation levels of corresponding sDMRs are either female-like or intermediate and become similar to those found in males by 16 weeks of age. Furthermore, although a limited number of sDMRs and sDEGs were tested in the present study, we still find regions where sex bias in expression precedes sex bias in methylation (male-biased *Elovl3* and *Hsd3b5,* and female-biased *Fmo3*).

If indeed, sex-biased expression is the signal for the establishment of sex-biased DNA methylation patterns, one would expect that not one, but several pathways that control gene expression in hepatocytes would contribute to sexual dimorphism in methylation levels. This would reconcile the multitude of factors, from testosterone and growth hormone signaling to specific TFs, that have been implicated to date in sex-biased methylation^5,8,11–13,15,25^. Therefore, we speculate that multiple signaling pathways affecting gene regulation, including steroid sex hormones, can modify methylation, at least at the early ages when methylation patterns are being established.

In the ESR1KO mice, changes in expression mirrored those in methylation in our panel of sDMRs. This was not the case in *Bcl6*-LKO mice. With the well-established role of BCL6 as a downstream effector of GH-signaling in liver and a transcriptional repressor of female-biased genes in males^12,14,43^, we expected to find demethylation of female-biased sDMRs mirroring the previously demonstrated upregulation of female-biased sDEGs in the *Bcl6*-LKO males^14^. Remarkably, we observed a dissociation between female-biased methylation and expression in the *Bcl6*-LKO mice with hypermethylation of female-biased sDMRs and upregulation of their proximal sDEGs. In combination with the female-like levels of *Bcl6* RNA in 8-week old hermaphrodites that show hypermethylation of male-biased sDMRs, these data suggest that BCL6 is involved in demethylation or the maintenance of low methylation levels at sDMRs. It is worth noting, that mice lacking two other effectors of GH-signaling, STAT5A and STAT5B, whose motifs partially overlap with the binding motif of BCL6^44^, show both hypo- and hypermethylation of sDMRs, suggesting the importance of STAT5 for both loss and gain of methylation^13^. Moreover, predominant hypermethylation of sDMRs located in STAT5-dependent active enhancer regions and downregulation of associated DEGs, but no discordance between methylation and expression has been reported for these mice^13^.

Based on the sum of data presented here, we propose that active gene transcription and availability of BCL6 are required for demethylation at certain sDMRs located within genic regions (Fig. 7d). It has been demonstrated that intronic TF-binding sites contribute to chromatin looping and gene regulation, potentially representing a novel class of regulatory elements^45,46^. Based on the BCL6 enrichment within actively transcribed genic regions, we speculate that it may bind such intronic regulatory elements and stabilize chromatin loops and contacts between gene promoters and distant enhancers facilitating continued gene expression. BCL6 binding may also restrict the spreading of DNA methylation from adjacent regions or recruit demethylation machinery to intronic/intragenic sDMRs.

Our working hypothesis outlining the distinct roles of AR, ESR1, and BCL6 is summarized below (Fig. 7d). AR, ESR1, and BCL6 activate the expression of male-biased genes in males. We propose that hypermethylation of male-biased intragenic sDMRs in ARKO, ESR1KO, or *Bcl6*-LKO males is the result downregulation of the sDMR-proximal genes. Based on data from other studies^47^, we speculate that in females, ligand-bound ESR1 may act as a transcriptional activator or a repressor, depending on the repertoires of interacting co-factors. For certain male-biased genes, ESR1 acts as a repressor and its loss results in modest increase in expression and hence hypomethylation of associated sDMRs. ESR1 also promotes expression of female-biased genes in wild type females and creates a permissive environment for BCL6 binding and sDMR demethylation. Consequently, in females, ESR1 enrichment at genic regions is accompanied by enrichment of BCL6. Loss of ESR1 dampens the transcription of female-biased genes and thereby prevents BCL6 binding, whereas loss of BCL6 does not have a major impact on expression but causes hypermethylation of sDMRs. In males, BCL6 binds promoter regions of female-biased genes, recruits repressive complexes, and hence promotes methylation of intragenic sDMRs, in agreement with previous studies^12,14^. BCL6 has no access to its intragenic binding sites due to lack of gene expression. Loss of BCL6 removes the expression block, hence female-biased genes are upregulated in *Bcl6*-LKO males. However, due to the absence of BCL6, intragenic female-biased sDMRs are not protected from methylation. Thus, we propose that ESR1 and AR influence DNA methylation through their impacts on gene expression, whereas BCL6 not only regulates expression, but also serves as a bridge between expression and demethylation at intragenic regions.

Our study has several limitations. Firstly, we focused on a panel of sDMRs selected for their association with sex-biased expression of their proximal genes. For technical reasons, sDMRs that overlap with repetitive sequences could not be tested by pyrosequencing. Three of the selected six sDMRs were located within introns, one in an exon, and two were intergenic, but in close proximity to sDEGs. Hence, our proposed model may be relevant for intragenic sDMRs, but other types of sDMRs may show different responses to the same mutations.

Secondly, use of global ARKO and ESR1KO, complicates data interpretation. One has to deconvolute the direct effects of AR or ESR1 on the liver genome from those stemming from their functions in other organs, including gonads or pituitary. In particular, the impacts on pituitary somatotropes and patterns of GH secretion, which are also implicated in the regulation of the liver epigenome, may be critical for sex-biased DNA methylation. However, the enrichment of AR or ESR1 near sDMRs in the mouse livers suggests that at least some of the effects are liver-specific (Table 1, Figs. 7a–c and S6).

Thirdly, in the DNA molecule, the cytosine may be either methylated or unmethylated. Hence different methylation levels in males and females reflect cellular mosaicism with respect to methylation. In contrast, gene expression analyses, unless tested using single-cell sequencing, FISH, or cell sorting, do not provide information about the mosaicism in gene expression. For example, in females the *Cyp7b1* has very low expression levels and its promoter region is about 70% methylated, whereas in males, expression is high and methylation levels are about 40%. This suggests that about 30% of liver cells in females and about 60% of liver cells in males carry an unmethylated *Cyp7b1* promoter and may express *Cyp7b1*. Analyses of gene expression in different zones of the liver reveal sex bias in the zonal distribution of hepatocytes expressing certain genes, including sex-biased genes from the CYP450 families^48^. Single-cell RNA-seq of male mouse liver shows higher expression of *Gstp1* and *Gstp2* near the portal vein and higher expression of *Cyp7b1*, *Hsd3b4*, *Hsd3b5*, and *Comt* near the central vein^49^. Interestingly, *Ar* RNA also shows a gradient with higher expression in hepatocytes adjacent to the central vein, whereas *Esr1* RNA levels are highest in hepatocytes adjacent to the portal vein^49^. In light of these data, analysis of different subpopulations of hepatocytes is necessary for understanding of how different factors, including sex hormones, influence sex-biased DNA methylation.

## Materials and methods

### Mouse strains and crosses

**C57BL/6J** mice were purchased from the Jackson Laboratory (Bar Harbor, Maine, USA). **B6.Y**^***TIR***^ mice were maintained in our colony (TT) by breeding of B6.Y^*TIR*^ males to C57BL/6J females. B6.Y^*TIR*^ males were crossed to wild-type C57BL/6J females to generate XY^*TIR*^ sex-reversed females, true hermaphrodites, and males, as well as XX females. Female offspring were genotyped using PCR amplification of the zinc finger protein on the Y (*Zfy)* sequence of DNA from ear punches, using the primers and conditions described in^50^. The list of genotyping primers is provided in Table S2.

Global **AR∆ZF2** male mice (referred to as ARKO) with an in-frame deletion of exon 3 encoding the 2nd zinc finger of the DNA binding domain were generated as described previously^27^. Mice were maintained on a congenic C57BL/6J background and WT male and female littermates were used as controls. Mice were housed in a conventional facility, with a 12-h light–dark cycle and standard chow and water provided ad libitum. All studies involving ARKO mice and littermates were undertaken with approval from the Austin Health Animal Ethics Committee.

**B6N(Cg)-Esr1**^***tm4.2Ksk***^**/J** mice^28^ (JAX stock #026176, referred to as ESR1KO) were purchased from the Jackson Laboratory (Bar Harbor, Maine, USA). ESR1KO heterozygous male and female mice were mated to produce homozygous ESR1KO mice. Genotype was determined by standard PCR (see Table S2 for primers).

***Bcl6-LKO mice***. Albumin promoter-*Cre* transgenic mice were obtained from Jackson Laboratory (Bar Harbor, ME). *Bcl6*-floxed mice were previously reported and distributed by RIKEN BioResource Research Center (Tsukuba, Japan)^51^. *Bcl6*-LKO mice were generated by mating *Bcl6*-floxed mice and albumin promoter-*Cre* transgenic mice. *Cre*-negative mice with *Bcl6* floxed alleles were designated as *Bcl6*-WT mice. *Cre*-positive mice with the *Bcl6*-floxed alleles were designated as *Bcl6*-LKO mice^14^. Animal experiments involving these strains were approved by the Institutional Animal Care and Use Committee of Tokai University (Japan, permit number: 173025). The genotyping primers for *Bcl6*-flox and *Cre* are provided in Table S2.

For the analysis of age-related methylation dynamics, livers were collected from prepubescent 4-week old and adult 8-week old C57BL/6J mice as well as from adult (8-week and 16-week old) offspring from F_1_(C57BL/6J x B6.Y^*TIR*^) crosses. For fetal liver collection, C57BL/6J mice were mated and the morning of detection of the vaginal plug was considered embryonic day 0.5 (E0.5). Embryos were collected at E14.5. Livers were isolated from embryos and used either for DNA or RNA extraction. Sex of the E14.5 C57BL/6J fetuses was determined using PCR of the sex-determining region Y (*Sry*) gene (Table S2).

For certain mutant mice, due to COVID-19 lockdowns, collections could not be performed at the same age, and the ages for different strains vary between 8 and 11 weeks. Livers from ESR1KO, *Bcl6*-LKO mice, and respective controls were collected at the age of 8 weeks. Livers from ARKO mice and controls were collected at 10–11 weeks.

All procedures were conducted in accordance with the guidelines set by the Canadian Council on Animal Care (Ottawa, Ontario, Canada) and were approved by the Animal Care Committee of the McGill University Health Center (Montreal, Quebec, Canada).

### DNA and RNA extraction

DNA from mouse livers was extracted either by using QIAamp Fast DNA TissueKit (Qiagen, NL) or a standard proteinase K/phenol/chloroform procedure. Total RNA was isolated using TRIzol Reagent (Thermo Fisher Scientific, MA, USA) according to the manufacturer's instructions and followed by purification using the RNeasy MinElute Cleanup Kit (Qiagen, NL).

### Pyrosequencing DNA methylation assays

DNA was treated with sodium bisulfite using EpiTect Bisulfite Kit (Qiagen, NL). Primers for differentially methylated regions (DMRs) (50–70 bp) were designed using the PyroMark Assay Design 2.0 Software (Qiagen, NL). PCR was conducted using the Hot Start Taq DNA polymerase (New England Biolabs, MA, USA). The list of primers is provided in Supplementary Table S3. Pyrosequencing was carried out using the PyroMark Q24 Advanced platform and PyroMark Q24 Advanced CpG Reagents (Qiagen, NL). Results were analyzed using the PyroMark Q24 Advanced software (Qiagen, NL).

### Gene expression assays

CDNA was synthesized using 1 μg of RNA, oligo dT 12–18 primers and Moloney Murine Leukemia Virus reverse transcriptase (M-MuLV RT) (Thermo Fisher Scientific, MA, US). Quantitative RT-PCR (qPCR) was performed using Power SYBR Green PCR master mix (Thermo Fisher Scientific, MA, USA) and Eco Real-Time PCR System (Illumina, CA, USA). Gene expression levels were normalized to the housekeeping gene ribosomal protein L19 (*Rpl19*). Primers for expression analysis were designed using Primer3 software and checked with Bisearch and IDT OligoAnalyzer (https://www.idtdna.com/pages/tools/oligoanalyzer). List of primers is provided in Table S4. Heatmap for expression differences between females and males was generated using ComplexHeatmap R package (version 2.6.2), and the circlize R package (version 0.4.12) was used to color the heatmap^52^.

### RNA-sequencing and analysis

Total RNA was extracted from the livers of 8-week old homozygous ESR1KO mice (5 females and 4 males) and WT controls (3 females, 3 males) using TRIzol Reagent (Thermo Fisher Scientific, MA, USA) and purified using the RNeasy MinElute Cleanup Kit (Qiagen, NL). Library preparation and paired-end sequencing using an Illumina NovaSeq6000 S4 sequencer in a single lane and a second top-up run for samples with low reads were performed by the McGill Genome Centre (Montreal, QC, Canada).

Differential expression analysis was performed using the GenPipes RNAseq pipeline (v.3.2.0)^53^. Reads were first trimmed and filtered for quality, before being aligned to the mouse reference genome (GRCm38) using STAR (v.2.7.3a)^54^. Transcript abundance was estimated using HT-Seq Count (v. 0.11.0)^55^. PC analysis was performed on the abundance data after doing a regularized log transformation using DESeq2^56^. Differential gene expression was determined using the DESeq2 (v.1.20.0)^56^ and EdgeR (v.3.22.5)^57^ packages. Data are deposited to the NCBI GEO repository as GSE174535.

### Motif enrichment analysis with HOMER

The genomic coordinates of male-biased, female-biased, or combined sDMRs were used to detect enrichments of previously known transcription factor binding sites with HOMER (v.4.9.1)^58^. An option “-len 8,10” in defining the target motif lengths (targeting motifs with length 8 bp and 10 bp) was applied and default parameters were used if not mentioned. The top 20 enriched motifs in each of the three sDMR groups were presented as heatmaps, where the color intensity denoted the enrichment level (in %) of motifs.

### Co-localization of ChIP sites with sex-phenotype associated sDMRs

Lists of sex-phenotype dependent sDMRs (generated by comparing XY females to XY males)^6^ were compared to ChIPseq data for AR, ESR1, and BCL6. The lists of sDMRs were filtered to remove any located on the sex chromosomes, leaving a total of 3847 autosomal sDMRs. Male-biased (n = 3414) and female-biased (n = 433) sDMRs were analyzed separately. Processed AR and ESR1 ChIP-seq liver data (AR in males, ESR1 in females) were retrieved from the Array Express (E-MTAB-805) ChIP dataset^30^, filtered to remove sites with a fold enrichment value of − 2, and coordinates were converted to the mm10 assembly. A total of 7850 autosomal AR and 31,235 autosomal ESR1 ChIP enriched regions were analyzed. Data for BCL6 ChIP peaks were retrieved from GEO (GSE31578)^12^. Female liver replicate 3 (GSM784032) and male liver replicate 2 (GSM784028) were used for the analysis, as they had similar numbers of peaks (22,851 and 20,994 on autosomes, respectively).

The nearest ChIP peaks to each sDMR were identified using the BEDOPs closest-features tool (tool version 2.4.35^59^). To generate a background baseline control, the sDMR coordinates were shuffled using BEDTOOLS shuffle (v2.29.2^60^) and then the nearest ChIP peaks were found. This was repeated 100 times for each comparison. The cumulative distribution of distances between ChIP peaks and sDMRs or randomly shuffled control were generated with the function *density* of the package stats (version 4.0.3) in R. The number of direct overlaps (minimum one base) and overlaps within 10 kb between sDMRs and ChIP peaks was determined by filtering the bedtools results files in R. Permutation analysis was performed by shuffling sDMR coordinates 500 times and running closest-features, as described above, counting the number within overlapping or within a 10 kb distance from the ChIP-seq peak.

### Statistical analyses

We used one-way or two-way ANOVA followed by post hoc analyses with Tukey’s or Sidak’s correction for multiple testing, respectively, to compare our groups using GraphPad Prism 8 software.

This study is reported in accordance with ARRIVE guidelines (https://arriveguidelines.org).

## Supplementary Information

Supplementary Information.

## References

[1] Engel N (2018). Sex differences in early embryogenesis: Inter-chromosomal regulation sets the stage for sex-biased networks. BioEssays.

[2] Rancourt RC, Schellong K, Tzschentke B, Henrich W, Plagemann A (2018). DNA methylation and expression of proopiomelanocortin (POMC) gene in the hypothalamus of three-week-old chickens show sex-specific differences. FEBS Open Bio.

[3] Hu J (2019). The epigenetic signature of colonizing new environments in anolis lizards. Mol. Biol. Evol..

[4] Laing LV (2018). Sex-specific transcription and DNA methylation profiles of reproductive and epigenetic associated genes in the gonads and livers of breeding zebrafish. Comp. Biochem. Physiol. A Mol. Integr. Physiol..

[5] McCormick H (2017). Isogenic mice exhibit sexually-dimorphic DNA methylation patterns across multiple tissues. BMC Genom..

[6] Zhuang QK (2020). Sex chromosomes and sex phenotype contribute to biased DNA methylation in mouse liver. Cells.

[7] Duncan CG (2018). Dosage compensation and DNA methylation landscape of the X chromosome in mouse liver. Sci. Rep..

[8] Reizel Y (2015). Gender-specific postnatal demethylation and establishment of epigenetic memory. Genes Dev..

[9] Conforto TL, Waxman DJ (2012). Sex-specific mouse liver gene expression: Genome-wide analysis of developmental changes from pre-pubertal period to young adulthood. Biol. Sex Differ..

[10] Waxman DJ, O'Connor C (2006). Growth hormone regulation of sex-dependent liver gene expression. Mol. Endocrinol..

[11] Lau-Corona D, Suvorov A, Waxman DJ (2017). Feminization of male mouse liver by persistent growth hormone stimulation: Activation of sex-biased transcriptional networks and dynamic changes in chromatin states. Mol. Cell Biol..

[12] Zhang Y, Laz EV, Waxman DJ (2012). Dynamic, sex-differential STAT5 and BCL6 binding to sex-biased, growth hormone-regulated genes in adult mouse liver. Mol. Cell Biol..

[13] Hao P, Waxman DJ (2021). STAT5 regulation of sex-dependent hepatic CpG methylation at distal regulatory elements mapping to sex-biased genes. Mol. Cell Biol..

[14] Chikada H (2018). Establishment and analysis of a mouse model that regulates sex-related differences in liver drug metabolism. Lab. Invest..

[15] Conforto TL, Zhang Y, Sherman J, Waxman DJ (2012). Impact of CUX2 on the female mouse liver transcriptome: Activation of female-biased genes and repression of male-biased genes. Mol. Cell Biol..

[16] Houle AM, Taketo T (1992). True hermaphrodites: An experimental model in the mouse. J. Urol..

[17] Eicher E, Washburn L, Whitney J, Morrow K (1982). Mus poschiavinus Y chromosome in the C57BL/6J murine genome causes sex reversal. Science.

[18] Coward P (1994). Polymorphism of a CAG trinucleotide repeat within Sry correlates with B6.YDom sex reversal. Nat. Genet..

[19] Chaboissier MC (2004). Functional analysis of Sox8 and Sox9 during sex determination in the mouse. Development.

[20] Sekido R, Lovell-Badge R (2008). Sex determination involves synergistic action of SRY and SF1 on a specific Sox9 enhancer. Nature.

[21] Park S, Zeidan K, Shin JS, Taketo T (2011). SRY upregulation of SOX9 is inefficient and delayed, allowing ovarian differentiation, in the B6.Y(TIR) gonad. Differentiation.

[22] Nagamine CM, Taketo T, Koo GC (1987). Studies on the genetics of tda-1 XY sex reversal in the mouse. Differ.; Res. Biol. Divers..

[23] Taketo-Hosotani T, Nishioka Y, Nagamine CM, Villalpando I, Merchant-Larios H (1989). Development and fertility of ovaries in the B6.YDOM sex-reversed female mouse. Development (Cambridge, England).

[24] Nagamine CM, Taketo T, Koo GC (1987). Morphological development of the mouse gonad in tda-1 XY sex reversal. Differentiation.

[25] Melia T, Waxman DJ (2020). Genetic factors contributing to extensive variability of sex-specific hepatic gene expression in Diversity Outbred mice. PLoS ONE.

[26] Sommars MA (2019). Dynamic repression by BCL6 controls the genome-wide liver response to fasting and steatosis. Elife.

[27] Notini AJ, Davey RA, McManus JF, Bate KL, Zajac JD (2005). Genomic actions of the androgen receptor are required for normal male sexual differentiation in a mouse model. J. Mol. Endocrinol..

[28] Hewitt SC (2010). Biological and biochemical consequences of global deletion of exon 3 from the ER alpha gene. FASEB J..

[29] Sugathan A, Waxman DJ (2013). Genome-wide analysis of chromatin states reveals distinct mechanisms of sex-dependent gene regulation in male and female mouse liver. Mol. Cell Biol..

[30] Li Z, Tuteja G, Schug J, Kaestner KH (2012). Foxa1 and Foxa2 are essential for sexual dimorphism in liver cancer. Cell.

[31] Ito S (2015). Novel sex-dependent differentially methylated regions are demethylated in adult male mouse livers. Biochem. Biophys. Res. Commun..

[32] Dkhil MA (2015). Epigenetic modifications of gene promoter DNA in the liver of adult female mice masculinized by testosterone. J. Steroid Biochem. Mol. Biol..

[33] Wainwright EN, Wilhelm D (2010). The game plan: cellular and molecular mechanisms of mammalian testis development. Curr. Top. Dev. Biol..

[34] Corbier P, Edwards DA, Roffi J (1992). The neonatal testosterone surge: A comparative study. Arch. Int. Physiol. Biochim. Biophys..

[35] Clarkson J, Herbison AE (2016). Hypothalamic control of the male neonatal testosterone surge. Philos. Trans. R. Soc. Lond. B Biol. Sci..

[36] Hau J, Svendsen P, Teisner B, Pederson GT, Kristiansen B (1981). Correlation between fetal weight and maternal serum levels of murine alpha-fetoprotein and quantitation of four molecular forms. Biol. Reprod..

[37] Savu L, Benassayag C, Vallette G, Christeff N, Nunez E (1981). Mouse alpha 1-fetoprotein and albumin. A comparison of their binding properties with estrogen and fatty acid ligands. J. Biol. Chem..

[38] De Mees C (2006). Alpha-fetoprotein controls female fertility and prenatal development of the gonadotropin-releasing hormone pathway through an antiestrogenic action. Mol. Cell Biol..

[39] Khosla S (2006). Editorial: Sex hormone binding globulin: Inhibitor or facilitator (or both) of sex steroid action?. J. Clin. Endocrinol. Metab..

[40] Laurent MR (2016). Sex hormone-binding globulin regulation of androgen bioactivity in vivo: Validation of the free hormone hypothesis. Sci. Rep..

[41] Sullivan PM, Petrusz P, Szpirer C, Joseph DR (1991). Alternative processing of androgen-binding protein RNA transcripts in fetal rat liver. Identification of a transcript formed by trans splicing. J. Biol. Chem..

[42] Jänne M, Hogeveen KN, Deol HK, Hammond GL (1999). Expression and regulation of human sex hormone-binding globulin transgenes in mice during development. Endocrinology.

[43] Yang H, Green MR (2019). Epigenetic programing of B-cell lymphoma by BCL6 and its genetic deregulation. Front. Cell Dev. Biol..

[44] Meyer RD, Laz EV, Su T, Waxman DJ (2009). Male-specific hepatic Bcl6: Growth hormone-induced block of transcription elongation in females and binding to target genes inversely coordinated with STAT5. Mol. Endocrinol..

[45] Panigrahi AK (2018). SRC-3 coactivator governs dynamic estrogen-induced chromatin looping interactions during transcription. Mol. Cell.

[46] Panigrahi A, O'Malley BW (2021). Mechanisms of enhancer action: The known and the unknown. Genome Biol..

[47] Hsu PY (2010). Estrogen-mediated epigenetic repression of large chromosomal regions through DNA looping. Genome Res..

[48] Saito K, Negishi M, Squires EJ (2013). Sexual dimorphisms in zonal gene expression in mouse liver. Biochem. Biophys. Res. Commun..

[49] Halpern KB (2017). Single-cell spatial reconstruction reveals global division of labour in the mammalian liver. Nature.

[50] Amleh A, Smith L, Chen H, Taketo T (2000). Both nuclear and cytoplasmic components are defective in oocytes of the B6.Y(TIR) sex-reversed female mouse. Dev. Biol..

[51] Kaji T (2012). Distinct cellular pathways select germline-encoded and somatically mutated antibodies into immunological memory. J. Exp. Med..

[52] Gu Z, Gu L, Eils R, Schlesner M, Brors B (2014). Circlize implements and enhances circular visualization in R. Bioinformatics.

[53] Bourgey M (2019). GenPipes: An open-source framework for distributed and scalable genomic analyses. GigaScience.

[54] Dobin A (2013). STAR: Ultrafast universal RNA-seq aligner. Bioinformatics.

[55] Anders S, Pyl PT, Huber W (2015). HTSeq–a Python framework to work with high-throughput sequencing data. Bioinformatics.

[56] Anders S, Huber W (2010). Differential expression analysis for sequence count data. Genome Biol..

[57] Robinson MD, McCarthy DJ, Smyth GK (2010). edgeR: A Bioconductor package for differential expression analysis of digital gene expression data. Bioinformatics.

[58] Heinz S (2010). Simple combinations of lineage-determining transcription factors prime cis-regulatory elements required for macrophage and B cell identities. Mol. Cell.

[59] Neph S (2012). BEDOPS: High-performance genomic feature operations. Bioinformatics.

[60] Quinlan AR, Hall IM (2010). BEDTools: A flexible suite of utilities for comparing genomic features. Bioinformatics.

