# Author Correction: Distinct roles of androgen receptor, estrogen receptor alpha, and BCL6 in the establishment of sex-biased DNA methylation in mouse liver

**DOI:** 10.1038/s41598-021-02512-8

**Published:** 2021-11-22

**Authors:** Najla AlOgayil, Klara Bauermeister, Jose Hector Galvez, Varun S. Venkatesh, Qinwei Kim-wee Zhuang, Matthew L. Chang, Rachel A. Davey, Jeffrey D. Zajac, Kinuyo Ida, Akihide Kamiya, Teruko Taketo, Guillaume Bourque, Anna K. Naumova

**Affiliations:** 1 Department of Human Genetics, McGill University, Montréal, QC Canada; 2 Canadian Centre for Computational Genomics, Montréal, QC Canada; 3 Department of Medicine, Austin Health, The University of Melbourne, Heidelberg, VIC 3084 Australia; 4 Department of Biochemistry, McGill University, Montréal, QC Canada; 5 Department of Molecular Life Sciences, Tokai University School of Medicine, 143 Shimokasuya, Isehara, Kanagawa 259-1193 Japan; 6 The Research Institute of the McGill University Health Centre, Montreal, QC H4A 1J3 Canada; 7 Department of Surgery, McGill University, Montreal, QC Canada; 8 Department of Obstetrics and Gynecology, McGill University, Montreal, QC Canada

Correction to: *Scientific Reports* 10.1038/s41598-021-93216-6, published online 02 July 2021

The original version of this Article contained an error in Figure 5 where the labels indicating the names of the tested loci were omitted.

The original Figure 5 and accompanying legend appear below. The original Article has been corrected.

**Figure 5:**
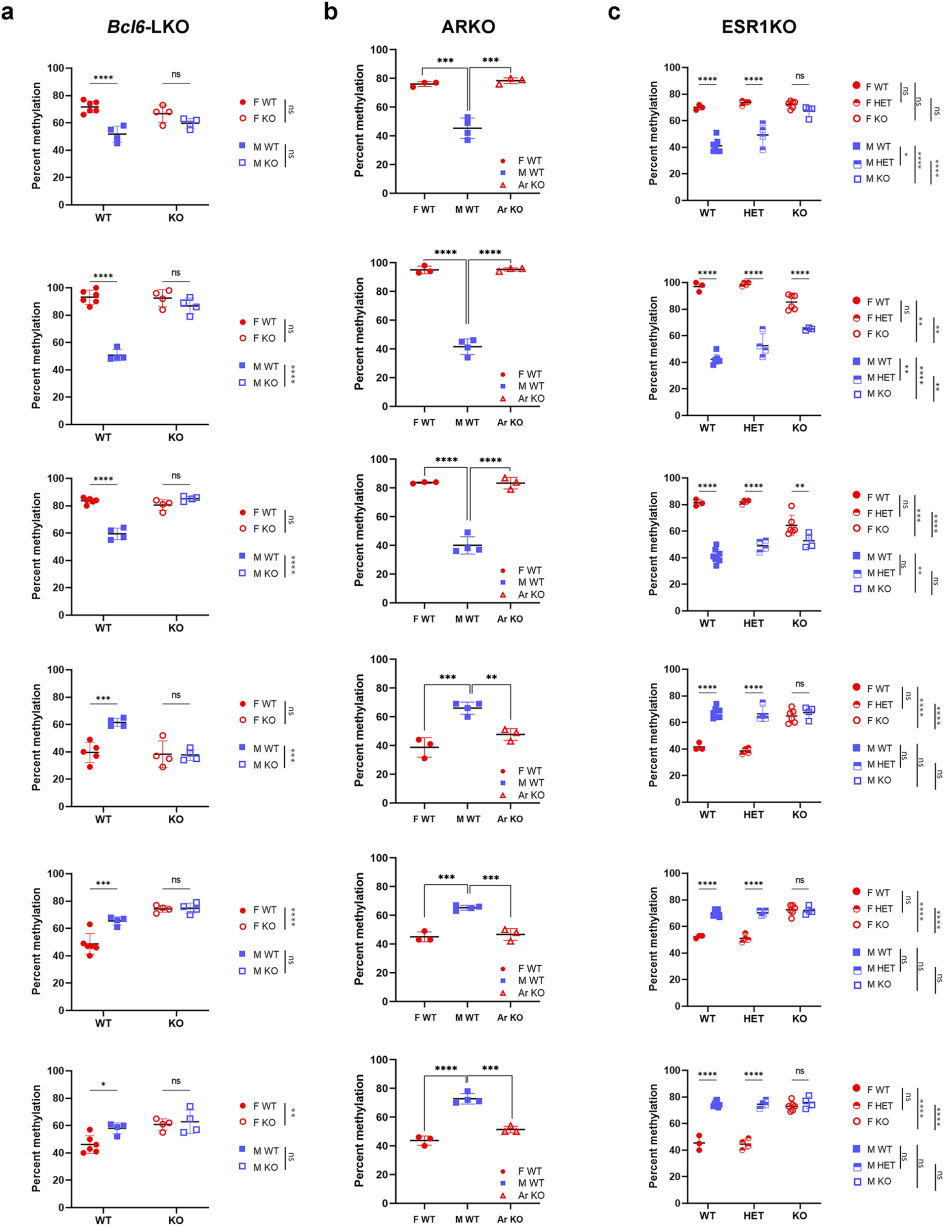
BCL6, AR, and ESR1 influence DNA methylation at sDMRs. (**a**) Methylation levels in male and female *Bcl6*-LKO (4 females, 4 males) and controls *Bcl6*-flox mice (6 females, 4 males); (**b**) Methylation levels in ARKO mice (with genetic and gonadal male sex and genital female sex) and control male and female littermates (3 females, 4 males, and 3 ARKO mice); (**c**) Methylation levels in wild type controls, heterozygous and homozygous ESR1KO mice (WT: 3 females, 7 males; HET: 4 females, 4 males, KO: 6 females, 4 males). Error bars show standard deviation. Statistically significant differences are shown with asterisks *P < 0.05, **P < 0.01, ***P < 0.001, ****P < 0.0001, ns: non-significant [two-way ANOVA, followed by multiple testing with Sidak’s correction (**a** and **c**); one-way ANOVA, followed by multiple testing with Tuckey’s correction (**b**)].

