# Supplementary material for: Distinct roles of androgen receptor, estrogen receptor alpha, and BCL6 in the establishment of sex-biased DNA methylation in mouse liver
Source: Sci Rep. 2021 Jul 2;11:13766. doi: 10.1038/s41598-021-93216-6 (PMC8253761; doi:10.1038/s41598-021-93216-6)
Supplement: Supplementary file 1 — Supplementary Information. [file 41598_2021_93216_MOESM1_ESM.pdf]

Table S1. Sex-biased differentially methylated regions (sDMRs) tested in our study and associated sex-biased differentially expressed genes (sDEGs) and non-biased proximal genes.

sDMR	Bias in methylation	Gene	Bias in expression	Fold difference [log2] F/M	Adjusted p-value [dseq]
Cyp7b1 (intron)	F>M	*Cyp7b1*	M>F	-2.92 (-2.7)	1.10E-13 (3.7E-12)
Gstp1 (distal enhancer, genic region)	F>M	*Gstp1*	M>F	-3.15 (-2.9)	8.80E-11 (7.7E-10)
		Gstp2	M>F	-1.25 (-1.3)	0.075 (0.046)
Hsd3b5 (intron)	F>M	*Hsd3b5*	M>F	-5.06 (-2.9)	1.40E-57 (1.7E-26)
		Gm12400	M>F	-2.28 (-5.06)	0.34 (0.028)
		Hsd3b3	M>F	-0.49 (-0.42)	0.044 (0.12)
		Hsd3b2	M>F	-0.72 (-0.74)	0.11 (0.082)
Comt (distal enhancer)	F>M	*Comt*	M>F	-0.86 (-0.71)	1.50E-05 (5.0E-04)
Esr1 (intron)	F>M	*Esr1*	F>M	0.75 (0.87)	0.024 (0.0034)
Elovl3 (intron)	F>M	*Elovl3*	M>F	-5.07 (-5.28)	4.7E-25 (1.0E-26)
Cux2 (intron)	M>F	*Cux2*	F>M	5.26 (4.99)	1.20E-18 (5.7E-202)
Cyp2b9 (exon-3'UTR)	M>F	*Cyp2b9*	F>M	6.67(6.66)	2.80E-181 (0)
		Cyp2b13	F>M	9.68 (9.4)	6.8E-24 (1.2E-33)
		Cyp2b10	F>M	4.32 (3.8)	2.30E-13 (3.60E-06)
		Cyp2a4	F>M	3.96 (4.2)	8.00E-12 (1.60E-142)
		Gm8902	F>M	4.18 (3.8)	9.20E-05 (0.0014)
		Rnf170-ps	F>M	6.49 (6.34)	1.0E-10 (4.80E-19)
		Vmn1r-ps86	F>M	5.79 (4.53)	9.50E-4 (0.42)
		Vmn1r184	F>M	4.24 (3.14)	3.80E-05 (0.058)
Fmo3 (intron)	M>F	*Fmo3*	F>M	8.82 (8.89)	6.00E-99 (1.4E-22)
		Gm37273	F>M	6.73 (6.50)	7.10E-24 (6.40E-17)
		Fmo2	F>M	1.67 (1.8)	1.50E-21 (1.30E-11)
		Fmo1	F>M	0.86 (0.91)	3.40E-06 (9.40E-09)
		Fmo6	F>M	1.2	0.9
		Fmo4	F>M	0.6 (0.54)	0.022 (0.014)
Aldh3b3 (CTCF site)	M>F	*Aldh3b3*	F>M	5.17 (5.4)	2.40E-36 (1.5E-24)
		Aldh3b2	F>M	3.99 (4.99)	0.0012 (3.70E-09)

Data from WGBS and RNA-seq datasets [6] comparing XX.F to XY.M and XY.F to XY.M. The results from the comparison between XY.F and XY.M are shown in parentheses. Positive log2 fold difference values show higher expression in females. Negative log2 fold difference values show higher expression in males. Functional annotations for the sDMRs are based on ENCODE data.

Table S2. List of genotyping primers

Gene	Forward primer	Reverse primer
Cre	AGGTGTAGAGAAGGCACTCAGC	CTAATCGCCATCTTCCAGCAGG
Bcl6-flox	CCATTCTCAGAAGATTATGGCAGA	CACACTATACATCAGAAAAGAATG
Esr1	ATCCCATGTGCTTGAGTGGT	CCACTTCTCCTGGGAGTCTG
Sry	GCAGGCTGTAAAATGCCACT	ATGCAGGTGGAAAAGCCTTA
Zfy	AAGATAAGCTTACATAATCACATGGA	CCTATGAAATCCTTTGCTGCACATGT

Table S3. List of pyrosequencing methylation assay primers

Gene symbol	Chr.	CG position (mm10)	Primer 1 (5'-3')	Primer 2 (5'-3') biotinylated	Sequencing primer (5'-3')
Aldh3b3	19	3,955,095	TTTTGGTGGTTGTAGATAGTGGT	TAATTACCCCCCCCCCTACAAT	GTAGATAGTGGTTGGTA
Comt	16	18,407,984	AAGGGGAAGGTGTTTTTAGTTGATAATG	TTCTCCAAACCCTCCACCACTTTCATA	GGTGTTTTTAGTTGATAATGT
Cux2	5	121,999,270	TAGGTATGGAATAGGATTTTATGTGTT	ATAATAAATAACTCTCACCACCTTTACT	ATGTGTTTTTAAAAGGTTAAAGAT
Cyp2b9	7	26,210,279	GGTAAGTTTTGTTGTTTTAAAGGATATTGA	ATAACACCTAACTCCCTCAC	AAATATTTTTAGTATATTAGATT
Cyp7b1	3	18,239,446	GGTTATAAGGTTTGTGATATGTTGTTA	ATTCTTAACCAACTCTCTAAATATACAAT	GGTTTGTGATATGTTGTTATAG
Elovl3	19	46,134,351	GGTAGTGTTTTTTGAGAGGTGAGGGATTAT	CCACACCCACACCCTAAAATTTCCAAATAT	AGGTGAGGGATTATAGT
Esr1	10	4,729,743	TTGGGGTTAATTATTTATTTGTGAGT	TCCCAAAAACACATTCCAAAAC	TGAGTTATTGGGTTGG
Fmo3	1	162,982,506	GATAAAGGTATATTTGTTTATGGATATGT	AATTACTCTCTAACCAAACAATTAAAC	ATTTTATTTTGTGAGGTTGAA
Gstp1	19	4,034,872	GTTTTGGTTGTTTTGGAATTTATTATGT	AAATTTCTCTCCTTAACCTCAATATTCT	ATTTATTATGTAAATTAGGTTGG
Hsd3b5	3	98,626,053	TTGTAGATATTGAATAGATATTAGGGAATT	CTTCCCCAACTTACTTCTTAATCATA	ATTGAATAGATATTAGGGAATTTT
Snrpn	7	60,005,146	TTGGTAGTTGTTTTTTGGTAGGAT	TCCACAAACCCAACTAACCTTC	GTGTAGTTATTGTTTGGGA
Xist	X	103,481,082	GTAATAGTTATGGGGTAGATTTTGGA	CTTAACCTCTAATTTAACCAACACTAA	ATTTAGTAGGTTTAGAGAAT

Table S4. List of expression primers

Gene	Forward primer	Reverse primer
Aldh3b3	ATGACCCTGTCCAGCCTTC	ACGGAGGCCATTAAGCTTCT
Ar	AGAATCCCACATCCTGCTCA	AAGTCCACGCTCACCATATG
Bcl6	CTTCCGGCACCTTCAGACT	CAGTTGGCTTTTGTGACGAA
Comt	TTATCCCCCAGCTGAAGAAG	ACATACGCCAGGAAGTCAGG
Cyp2b9	TGAGCACTTTCTAGATGCCAAT	GGCAATGCTTTCACCAAGAC
Cyp2b13	AGCTCTCCATGACCCACAGT	GGAGGATGGACGTGAAGAAA
Cyp7b1	GCCCTCTTTCCTCCACTCAT	CCTCCTTTGAAAAACGTGCT
Cux2	CCCACTCGGGTCAAAGTC	GCTGCTCTCCTTCCAACTCA
Elk4	CTGACTCCGAGCCCCTTG	AGTGAACGGGCCATGACTG
Elovl3	TTCTCTTTCTTCTCAGCAAGGT	GTGGTACCAGTGGACAAAGA
Esr1	CAGACACTTTGATCCACCTGA	CGTTCTTGCATTTCATGTTGTAG
Esrra	GGCCACTCTCTGTGACCTTT	CACTCTGCAGTACTGACATCTGG
Ets1	GGAATTCAAGCTTTCTGACCCA	CCACGGCTCAGTTTCTCATA
Fmo2	TTGCCTTCGGAGACGACTAT	TGCAGTATCTGACTCTGGCTTT
Fmo3	TGATGAGAAAATGGGGGAAA	GCTTTGCACCAATGAAGGAG
Foxa1	ACTGTGAAGATGGAAGGGCA	CCGGAGTTCATGTTGCTGAC
Foxa2	AGCCGTGAAGATGGAAGGG	GGCGTTCATGTTGCTCACG
Foxa3	GCTCAGTGAAGATGGAGGCT	ATGGTGGGCACAGGATTCA
Gabpa	AAGAACAAGCCTACCATGAAC T	ACACAAATCTCTTGCCTTGAACT
Ghr	TGAAGGGATGGATAATTCTGGAG	TCTCAATGAGTACACTGGACAG
H19	ACTACCTGCCTCAGGAATCTG	TGGGTGGGTGCTATGAGTC
Hnf4a	GTTGCTAACACGATGCCCTC	GCTGTGGAGTCTCGGGAG
Onecut1	AGACCTTCCGGAGGATGTG	TTCCCGTGTTCTTGCTCTTT
Rpl19	GATCATCCGCAAGCCTGTGA	GCATCCGAGCATTGGCAGTA
Sox6	GGATTGGGGAGTACAAGCAA	CACCTGTTCCTGTGGTGATG
Stat5b	CTCCTTCCCCAGTCGTGTG	TCCAGATCGAAGTCCCCATC
Thrb	GCCGTCCTGCTAATGTCT	TGGTGCTTCCGGTAATTGAT

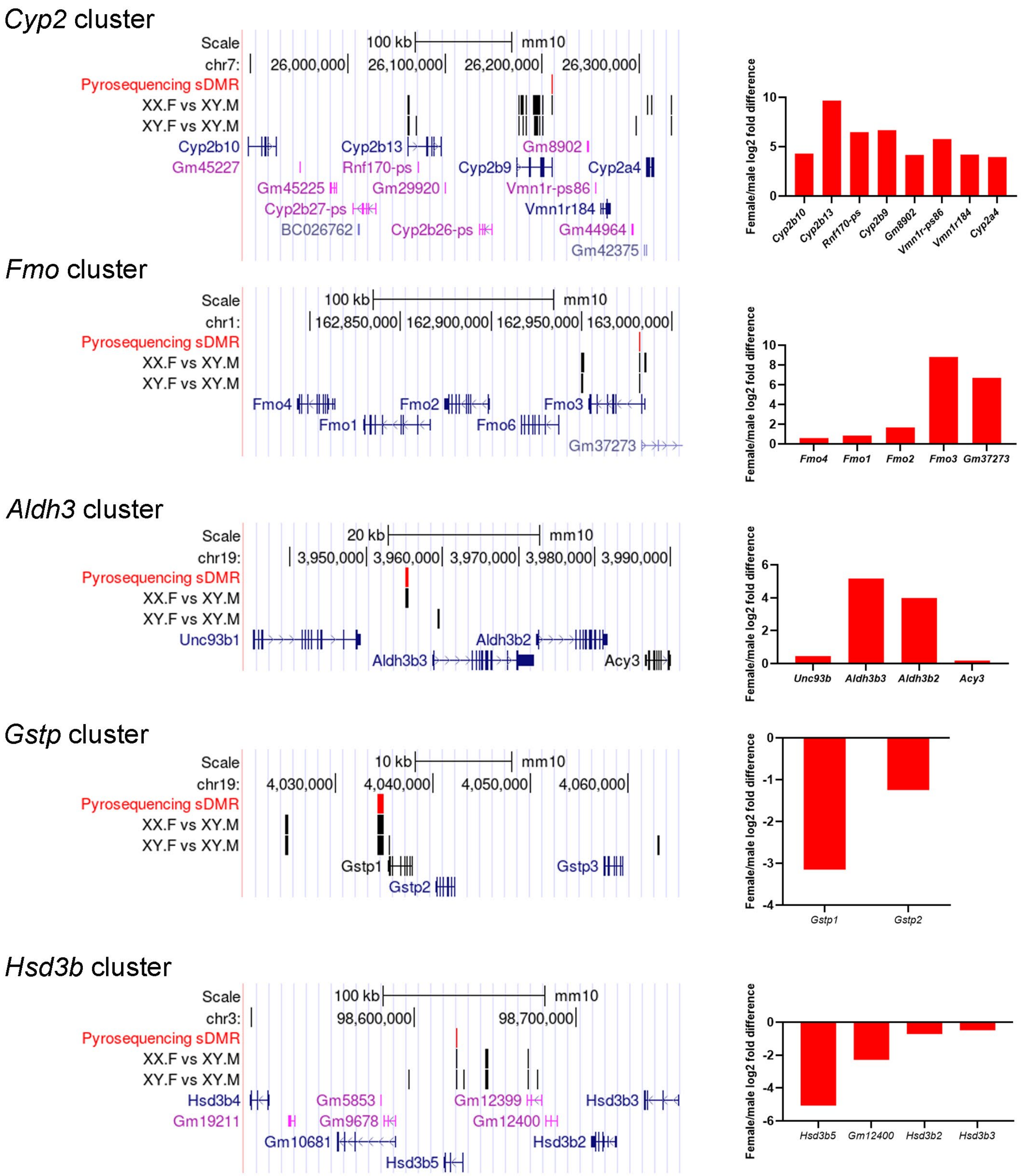

Figure S1. SDMRs associated with sDEGs clusters. All tracks are shown in the context of the UCSC genome browser (mm10). The ‘Pyrosequencing sDMR’ track shows the sDMR used for pyrosequencing methylation analysis. The ‘XX.F vs XY.M’ and ‘XY.F vs XY.M’ tracks show the locations of sDMRs based on WGBS data from [6]. On the right, log2 fold difference in expression between XX.F and XY.M is shown, based on RNA-seq data from [6]. Positive values indicate higher expression in females.

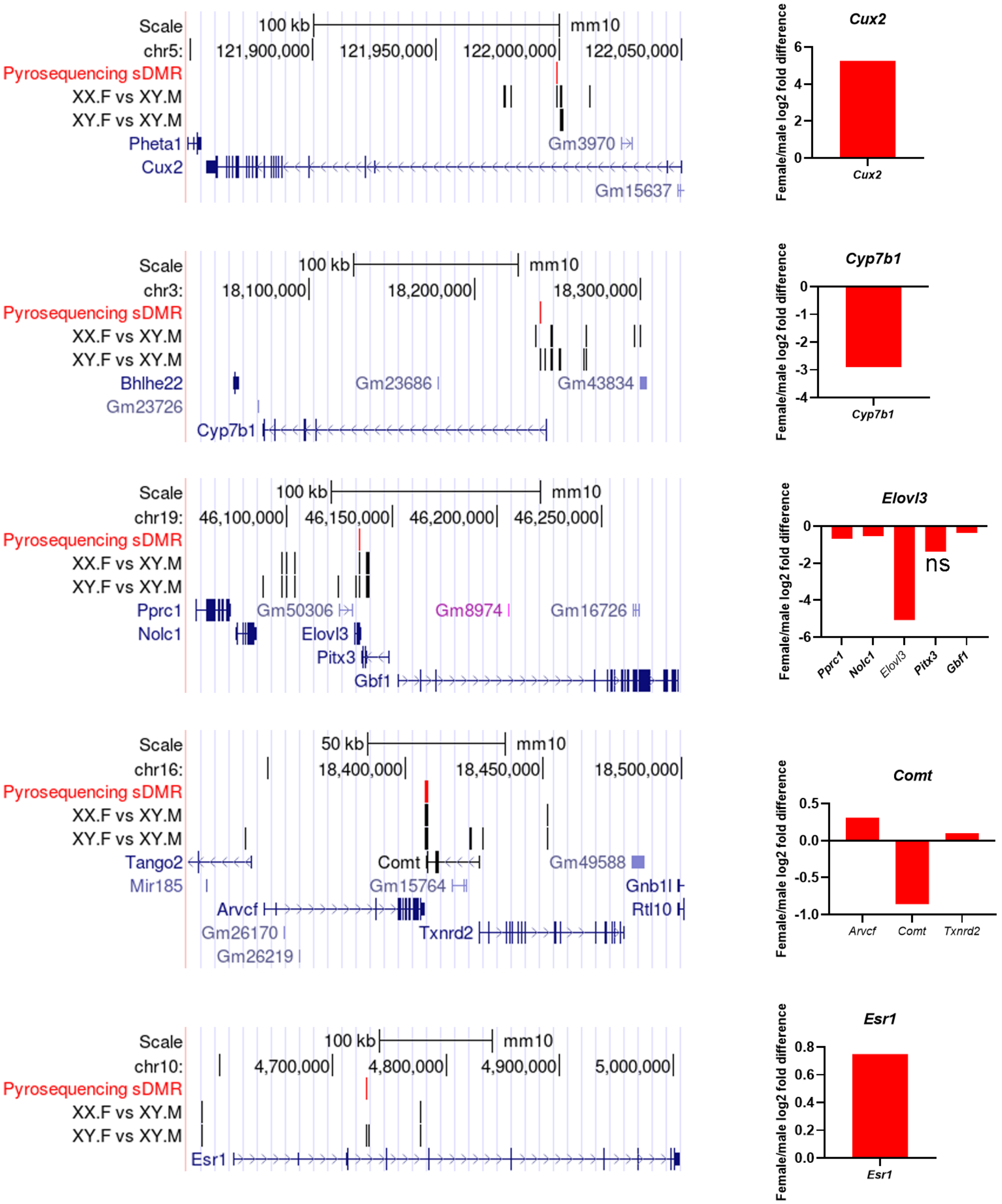

Figure S2. SDMRs associated with a single sDEG. All tracks are shown in the context of the UCSC genome browser (mm10). The 'Pyrosequencing sDMR' track shows the position of the sDMR tested in pyrosequencing methylation assays. The 'XX.F vs XY.M' and 'XY.F vs XY.M' tracks show the positions of sDMRs based on WGBS data from [6]. On the right, log2 fold difference in expression between XX.F and XY.M, based on RNA-seq data from [6]. Positive values indicate higher expression in females.

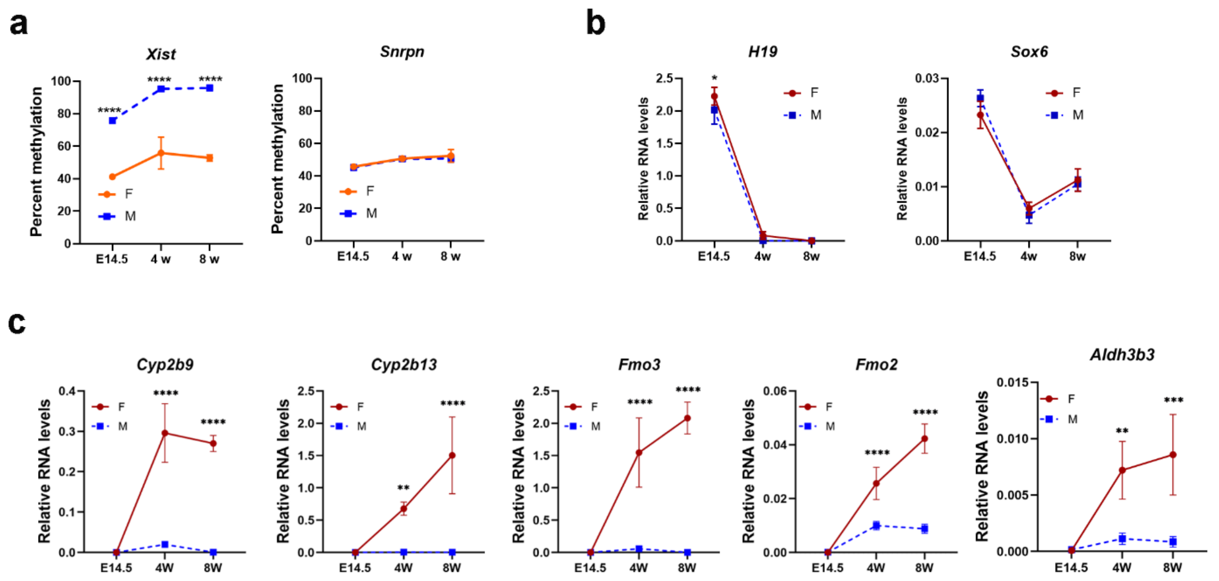

Figure S3. Developmental dynamics of DNA methylation and gene expression.

a. Methylation levels of *Xist* and *Snrpn* promoters at different ages. **b.** Developmental expression profiles of *H19* and *Sox6*. **c.** Developmental expression profiles of female-biased sDMRs-proximal sDEGs. All expression levels are normalized to *Rpl19*. Error bars show standard deviation. Statistically significant differences are shown with asterisks * P<0.05, ** P<0.01, *** P<0.001, **** P<0.0001 (two-way ANOVA followed by multiple testing with Sidak's correction).

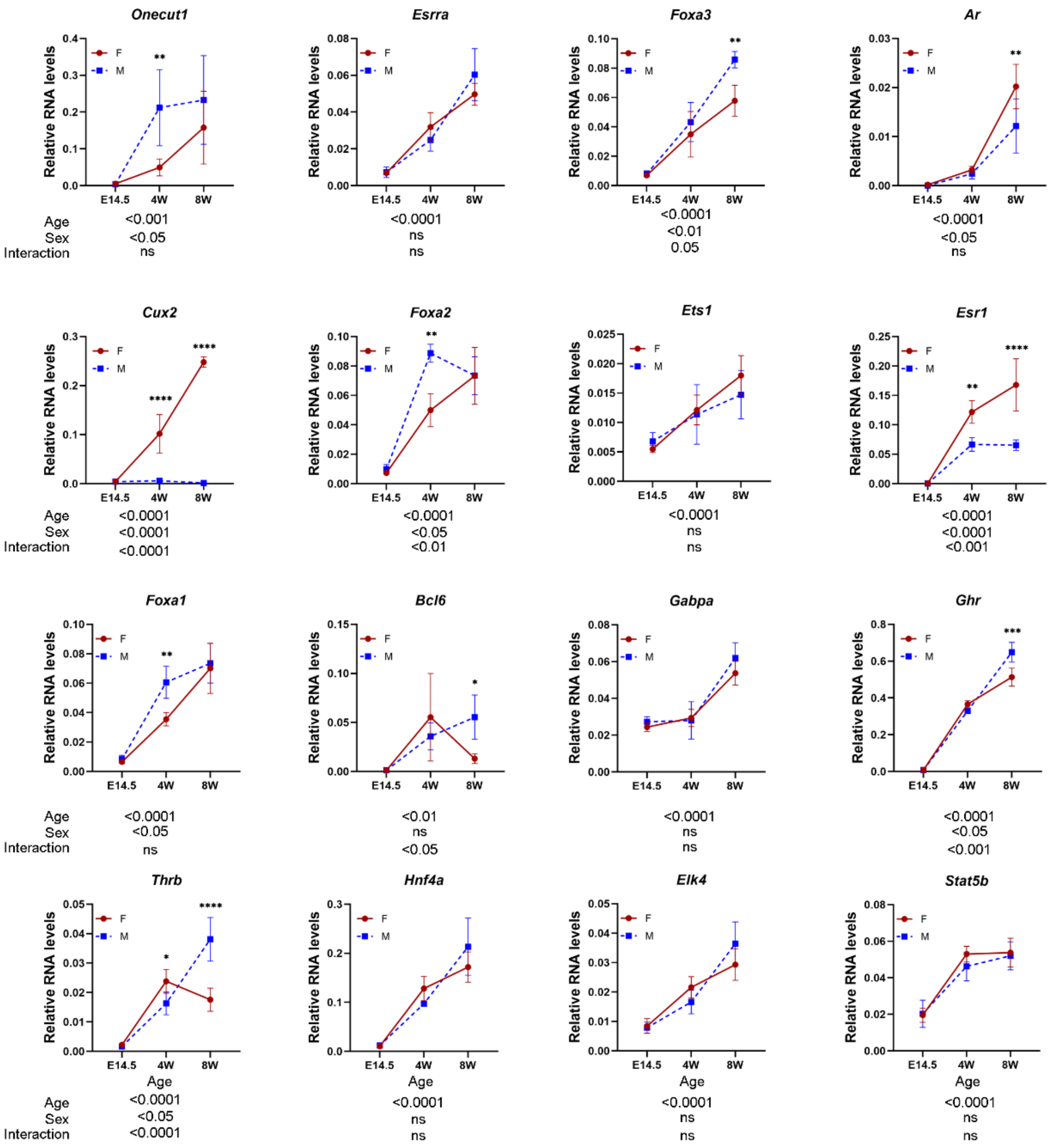

Figure S4. Developmental expression profiles of genes encoding transcription factors.
All expression levels are normalized to *Rpl19*. Results of two-way ANOVA testing of the impact of sex and age on expression shown below the diagrams. Error bars show standard deviation. Statistically significant differences are shown with asterisks * P<0.05, ** P<0.01, *** P<0.001, **** P<0.0001, ns: non-significant (two-way ANOVA followed by multiple testing with Sidak's correction).

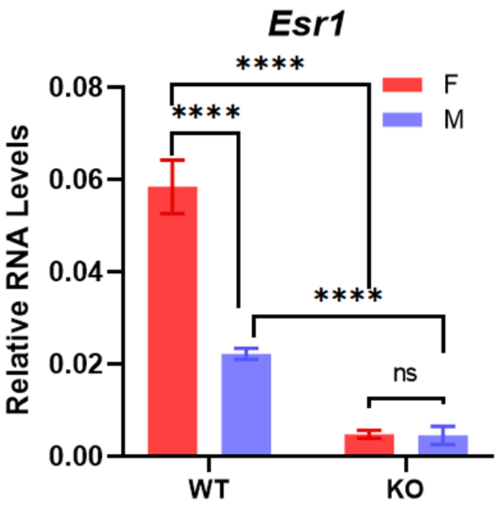

Figure S5. Confirmation of low expression of *Esr1* in mutant ESR1KO mice.
Expression levels are normalized to *Rpl19*.Error bars show standard deviation. Statistically significant differences are shown with asterisks **** P<0.0001, ns: non-significant (two-way ANOVA followed by multiple testing with Sidak's correction).

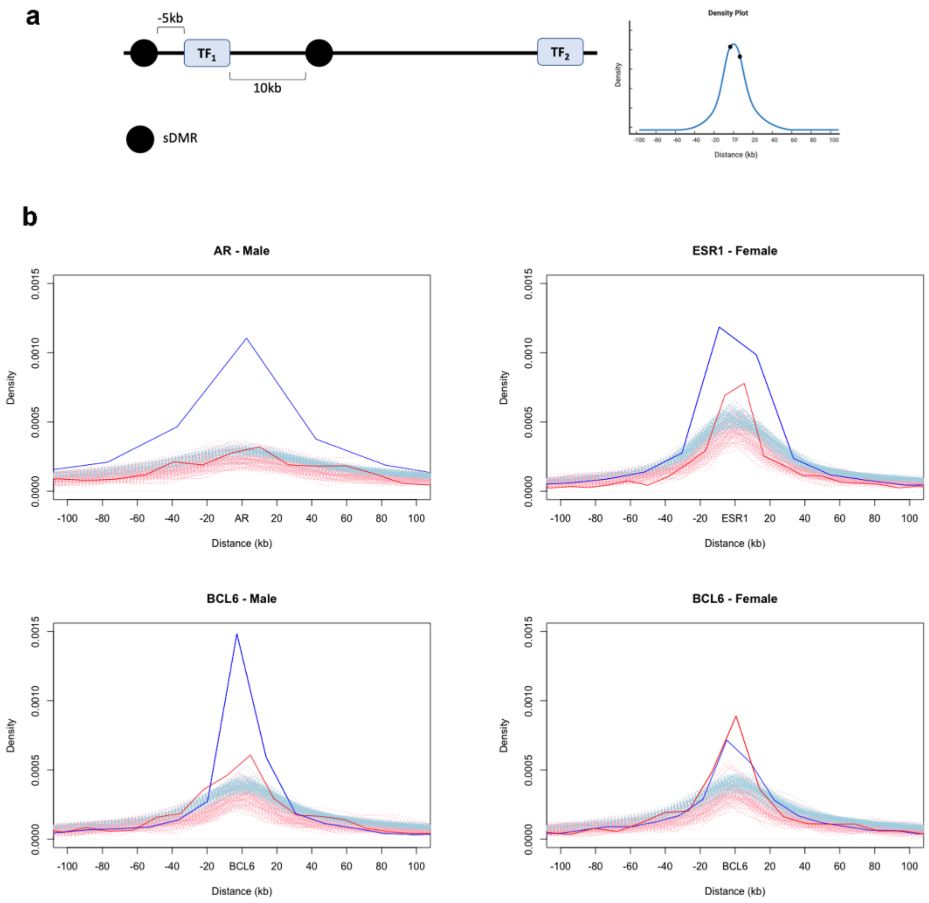

Figure S6. Distribution of distances from the TF-binding site to the closest sDMR.
a. Schematic shows how plots were generated. The distance between either end of the sDMR and the nearest ChIP-enriched regions was found, then a density plot was generated for each male-biased and female-biased sDMRs, relative to each AR-, ESR1-, or BCL6-enriched region in male or female liver. **b**. Density of sDMRs relative to AR ChIP peaks in male liver (left top panel); BCL6 ChIP peaks in male liver (left bottom panel); ESR1 ChIP peaks in female liver (right top panel), and BCL6 ChIP peaks in female liver (right bottom panel). The x-axis shows distance from the TF-enriched site, the y-axis shows density of sDMRs. Male-biased sDMRs – solid blue line, female-biased sDMRs - solid red line, dashed lines show 100 permutations for the male-biased (blue) or female-biased (red) sDMRs.
